# Connecting structure to function with the recovery of over 1000 high-quality metagenome-assembled genomes from activated sludge using long-read sequencing

**DOI:** 10.1038/s41467-021-22203-2

**Published:** 2021-03-31

**Authors:** Caitlin M. Singleton, Francesca Petriglieri, Jannie M. Kristensen, Rasmus H. Kirkegaard, Thomas Y. Michaelsen, Martin H. Andersen, Zivile Kondrotaite, Søren M. Karst, Morten S. Dueholm, Per H. Nielsen, Mads Albertsen

**Affiliations:** Center for Microbial Communities, Department of Chemistry and Bioscience, Aalborg University, Aalborg, Denmark

**Keywords:** Metagenomics, Water microbiology, Microbial communities

## Abstract

Microorganisms play crucial roles in water recycling, pollution removal and resource recovery in the wastewater industry. The structure of these microbial communities is increasingly understood based on 16S rRNA amplicon sequencing data. However, such data cannot be linked to functional potential in the absence of high-quality metagenome-assembled genomes (MAGs) for nearly all species. Here, we use long-read and short-read sequencing to recover 1083 high-quality MAGs, including 57 closed circular genomes, from 23 Danish full-scale wastewater treatment plants. The MAGs account for ~30% of the community based on relative abundance, and meet the stringent MIMAG high-quality draft requirements including full-length rRNA genes. We use the information provided by these MAGs in combination with >13 years of 16S rRNA amplicon sequencing data, as well as Raman microspectroscopy and fluorescence in situ hybridisation, to uncover abundant undescribed lineages belonging to important functional groups.

## Abstract (web summary)

Microbes play key roles in wastewater treatment. Here, Singleton et al. use long-read and short-read sequencing to recover 1083 high-quality metagenome-assembled genomes from 23 wastewater treatment plants, and combine this information with amplicon data, Raman microspectroscopy and FISH to reveal functionally important lineages.

## Introduction

Since the first metagenome-assembled genomes (MAGs) were recovered in 2004^1,2^, thousands of MAGs have shed light on the myriad of important functions of bacteria and archaea across the world’s ecosystems. By taking advantage of cheap short-read sequencing, compute power, and new algorithms, the recovery of MAGs has increased exponentially, to enable the recovery of over 100,000 MAGs in a single study^3^. However, MAG quality has not improved to the same extent and there are increasing concerns regarding the quality of reference databases and the validity of research based on them^4,5^. The minimum information about a MAG (MIMAG) standard was introduced to unify the field’s reporting standards, stating that high-quality (HQ) draft MAGs for bacteria and archaea must be >90% complete, <5% contaminated, and, importantly, include the full-length 16S, 23S, and 5S rRNA genes, and >18 tRNA genes^6^. Under the MIMAG standards, only a very limited number of HQ draft MAGs have been recovered from any study to date^3,7–9^. HQ reference databases are required to confidently investigate the structure and function of complex microbial communities in natural or engineered ecosystems.

Wastewater treatment and resource recovery are subject to the increasing pressures of human population growth and the demands for sustainability, human health, and reduced environmental impact. Microorganisms underpin wastewater treatment processes, from organic matter degradation and bioenergy generation to the removal of contaminants and recovery of nutrients such as nitrogen and phosphorus^10,11^. Activated sludge (AS) is the most important system by volume worldwide for wastewater treatment and the functional capacity and quality of treatment is entirely dependent on the balance of the bacterial taxa within the AS biomass. Determining the structure and potential function of microbial communities in full-scale systems is central to tracking wastewater treatment efficiency and consolidating changes in structure with changes in the system, and for carrying out informed management of the communities^12^. The collection of full-length 16S rRNA genes from Danish wastewater treatment plants (WWTPs) in the microbial database for AS (MiDAS3 database) enables the structure (i.e., taxonomy and abundances) of wastewater bacteria to be determined and monitored using 16S rRNA gene amplicon data^13^. However, most of the abundant populations in Danish WWTPs are undescribed and have unknown functions^13^. MAGs are required to determine their functional potential, although further experiments are needed to confirm identified functions.

Although hundreds or thousands of MAGs have been recovered from AS systems through private and public datasets^9,14^, very few of these are of HQ. HQ MAGs are beginning to be recovered from these systems^15^ and are needed to solve challenges in the treatment process. For example, an overgrowth of particular microbial morphotypes can lead to solid–liquid separation problems during the settling phase^16^ and the absence of functional groups can lead to poor nutrient recovery^17^.

**Fig. 1:**
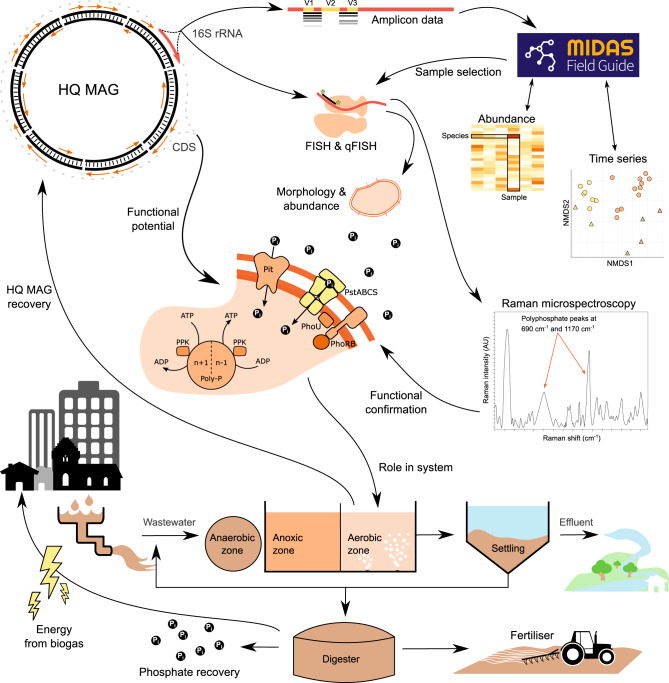
Conceptual overview of the value of HQ MAGs in linking structure to function. HQ MAGs with full-length 16S rRNA genes are recovered from a full-scale AS sample, allowing linkage to the abundance and time-series data of MiDAS, and informing sample selection for further experiments. MAG and MiDAS full-length 16S rRNA gene sequences facilitate creation of lineage-specific FISH probes. Abundance is confirmed with quantitative FISH (qFISH), and morphology and location in the floc is determined. The coding sequences (CDSs) provide information on functional potential, such as the presence of phosphate accumulation enzymes (e.g., PstABCS, Pit, and PPK), allowing for the selection of novel species that may belong to certain functional guilds. Specific potential pathways, such as polyphosphate accumulation, can be experimentally determined with Raman microspectroscopy in combination with FISH and the information gained from the full-length 16S rRNA gene. This leads to confirmation of the population’s role in the AS system and uncovers targets for investigation into improved resource recovery and effective wastewater treatment.

Here we demonstrate the combined use of long- and short-read sequencing for the high-throughput production of HQ MAGs from complex microbial communities. We also present the benefits of HQ MAGs, by linking genomic functional potential to the comprehensive MiDAS3 16S rRNA gene database, with >13 years of amplicon data, used to cost-effectively monitor WWTP microbial communities in Denmark^13,18^ (Fig. 1). This approach enabled us to target, visualize, and experimentally confirm the metabolic potential of an abundant, yet uncharacterized, genus widespread in Danish WWTPs (Fig. 1).

## Results and discussion

### Recovery of HQ MAGs

Taking advantage of the recent gains in affordable high-throughput long-read sequencing using the Oxford Nanopore PromethION platform, we combined 1 Tbp long-read (Oxford Nanopore) and 0.9 Tbp short-read (Illumina) data, and recovered 3733 medium quality (MQ) to HQ MAGs from across 23 Danish WWTPs (Supplementary Data 1 and 2). Of these MAGs, 1145 meet the MIMAG standard of HQ draft genomes with >90% completeness and <5% contamination, with 19 having circularized closed MAGs (CMAGs) (Supplementary Data 3). Full-length rRNA genes are usually missing in MAGs due to the difficulties associated with assembling conserved and repetitive regions using short-read sequences^19^. The combination of long- and short-read sequencing methods used here enabled 91.3% (1045 out of 1145) of the near-complete MAGs to encode full-length 16S rRNA genes in addition to full-length 23S and 5S rRNA genes.

**Fig. 2:**
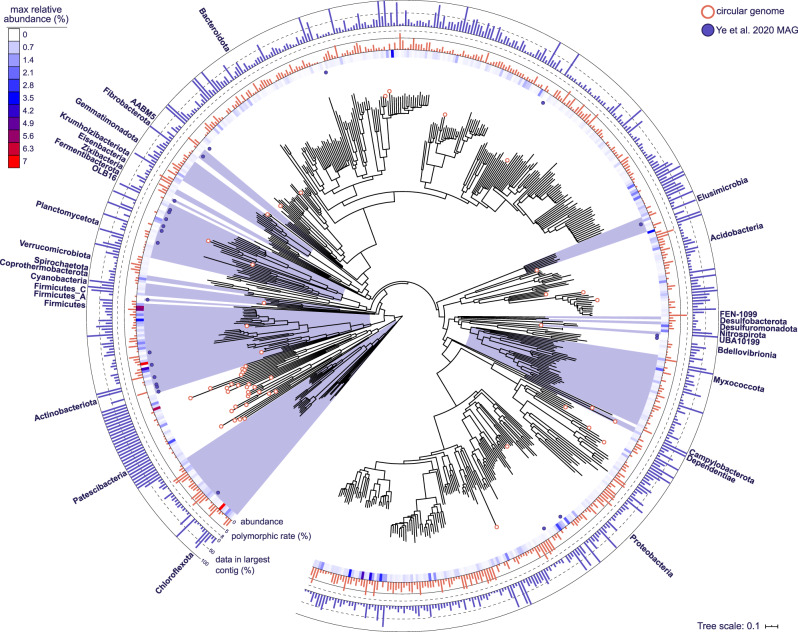
Phylogenetic bacterial genome tree showing the diversity, maximum abundance, and contiguity of recovered species. The tree is based on the concatenated alignment of 120 single-copy marker gene proteins using GTDB-Tk. The 578 HQ bacterial species representatives are shown, with phyla labeled. Circular genomes are indicated at the tips using a white filled circle. HQ MAGs from Ye et al.^9^ are indicated by the purple circles. The maximum relative abundance of the MAG across the 69 WWTP metagenomes is indicated by the heatmap. Polymorphic rate is indicated by the red bar chart and percentage of data incorporated in the longest contig within the MAG is indicated in the blue bar chart. Additional information on the MAGs is presented in Supplementary Data 3.

A further 38 CMAGs were identified and included in the HQ set, despite not meeting the 90% completeness threshold (Supplementary Data 3 and Fig. 2). These MAGs are likely complete, as they were circularized and most belong to recognized streamlined or reduced genome groups, such as the bacterial lineages Patescibacteria (Candidate Phyla Radiation) and Dependentiae^20^. However, three MAGs were from phyla not commonly associated with reduced genomes—the Proteobacteria (2 MAGs) and UBA10199 (1). A streamlined single contig genome was recently recovered for a Proteobacteria^7^ and our recovery of CMAGs for a novel Micavibrionales (2 Mbp) and Burkholderiales (0.9 Mbp) provides additional evidence of genome reduction in some members of this phylum. The group UBA10199 is undescribed and comprises a collection of only a few incomplete MAGs (24 in GTDB Release 04-RS89^21^). We believe the addition of our 38 small genome CMAGs will be valuable in revising single-copy marker gene-based completeness estimates for these lineages, which currently under-report completeness by up to 40% (Supplementary Data 3)^5,22^. In total, 57 CMAGs were recovered, nearly doubling the number of CMAGs in the public domain (62 reported by Chen et al. 2020 for September 10, 2019^5^).

All 1083 MAGs in the HQ set encode full-length 16S rRNA, 23S rRNA, 5S rRNA, and >18 tRNA genes, and had polymorphic site rates ranging from 0 to 7.7% (average 2%, Fig. 2 and Supplementary Data 3). Higher polymorphic site rates are suggestive of more chimeric population genomes and greater strain heterogeneity^3^.

### MAG diversity and community representation

**Fig. 3:**
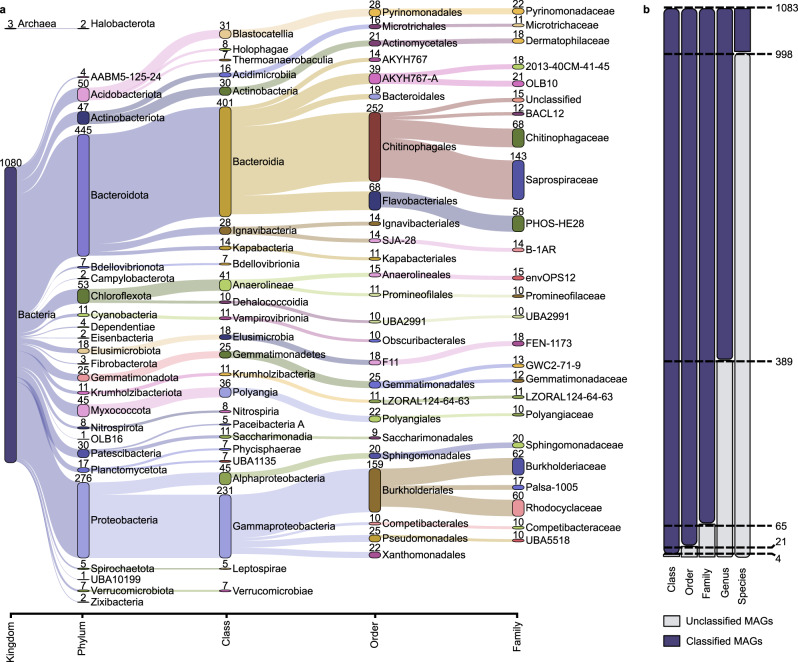
MAG recovery information across taxonomic levels. **a** Sankey based on assigned taxonomy showing the novel populations at different phylogenetic levels, with the top 25 taxa shown at each level. Numbers indicate the number of MAGs recovered for the lineage. **b** Total MAGs unclassified by GTDB-Tk at each taxonomic level.

The 1083 HQ MAGs represent 578 different bacterial and 3 different archaeal species (95% average nucleotide identity (ANI) clustering) (Fig. 2 and Supplementary Data 3). Phylogenetic analyses uncovered high taxonomic diversity from across 32 phyla, with the majority of MAGs attributed to the Bacteroidota (445 MAGs), Proteobacteria (276), and Chloroflexota (53). Genomes for uncharacterized phyla were also recovered, including AABM5-125-24 (4 MAGs), Krumholzibacteriota (11), and the Patescibacteria (30). An additional 317 Patescibacteria MAGs were recovered in the MQ set; many of these are likely to be near-complete with 76 comprising ≤5 contigs. An average of 44% and 30.9% of the populations in the metagenomes were successfully assembled or binned, respectively, based on analyses of the HQ MAG single-copy ribosomal protein sequences (Supplementary Fig. 1 and Supplementary Data 4). Clustering the markers at 89% ANI, representing approximate genus-level clusters^8^, showed that we have recovered a HQ MAG representative for the majority (58.4%) of the genera present in the sample metagenomes (Supplementary Data 4). This suggests we have created an HQ reference database (i.e., the MiDAS genome database) incorporating a large proportion of the lineages central to the AS process, which is an important step towards producing representative system-level databases. Coverage data based on mapping metagenome reads to the 581 species supported the recovery estimates, with an average of 27.2% of sample metagenomes mapping at >95% sequence identity and >75% alignment (Supplementary Data 5). Most MAGs had a relative abundance of ≥0.1% in at least one sample (515 species), while 66 MAGs represent consistently low relative abundance species (Supplementary Data 3 and 6, and Fig. 2). Close to all of the MAGs (998 or 92%) represent populations undescribed at the species level or at higher ranks (Fig. 3a, b and Supplementary Data 3).

Populations highly abundant in the metagenomes but not successfully binned mainly belonged to the Anaerolinea (Chloroflexota), Gamma- and Alphaproteobacteria (Proteobacteria), and Bacteroidia (Bacteroidota) (Supplementary Fig. 2). One specific Anaerolinea taxonomic unit, classified within the family envOPS12 and likely within *Ca*. Villigracilis, was consistently abundant yet unbinned across the metagenomes based on analysis of the ribosomal protein marker genes (Supplementary Fig. 3). This lineage was traced back to the MAG data and was found in two highly contaminated and fragmented bins (192% and 250% contaminated, 86% and 95% strain heterogeneity, >500 contigs). These bins could not be manually refined due to high strain heterogeneity, which was confirmed by the presence of up to five copies of highly similar single-copy ribosomal proteins in both bins (Supplementary Data 7). The variation in sequences in the single-copy ribosomal protein genes confirmed the presence of multiple sub-populations of the *Ca*. Villigracilis population in each sample (Supplementary Fig. 4). This strain heterogeneity likely caused well-known downstream problems in sequence assembly and binning^23^, which remains a challenge even when employing long-read sequencing^24^.

We compared our HQ MAGs to the largest AS MAG study to date by Ye et al.^9^. The authors recovered 2045 MAGs with a quality score (completeness − 5× contamination) ≥ 50 from 57 samples and 57 public datasets (114 metagenomes, including 16 metagenomes from Denmark). Of the Ye MAGs, 31% (644) met the MIMAG HQ completeness and contamination threshold (>90% completeness, <5% contamination) (Supplementary Data 8), but only 1.2% (25 MAGs) were of HQ according to the MIMAG standards and encoded the rRNA genes (Supplementary Data 9 and Fig. 2). After 95% ANI clustering with the HQ species representatives of this study, the 25 HQ Ye MAGs were found to belong to 24 species from predominantly the Planctomycetota (6 MAGs) and Actinobacteriota (6 MAGs) phyla (Supplementary Data 10). Three species, *Ca*. Microthrix parvicella and two MAGs belonging to uncharacterized lineages in the Elusimicrobia and Actinobacteriota, were represented by a better quality MAG from our set (Supplementary Data 10). The benefits of using long-read sequencing to improve both MAG quality and quantity are clear, as we recovered over 40× more HQ MAGs from fewer (23 vs. 114) metagenomes and a similar amount of data (830 Gb long-read, 922 Gb short-read vs. 1352 Gb). Using this method for large-scale recovery efforts in similarly complex environments, such as soil and the human gut, would lead to huge increases in the quality of all associated MAG reference databases.

However, it is important to acknowledge the widespread limitations of MAGs that represent chimeric population bins (not strains), are recovered in pipelines without individual detailed manual curation, and are affected by sequencing platform errors and low coverage regions^5,15^. Although still imperfect, this comprehensive collection of HQ MAGs provides the first links between 16S rRNA gene-based studies and the complete gene repertoires for many uncharacterized organisms in AS.

### Functional guilds central to wastewater resource recovery

The recovered MAGs facilitated analysis into the functional potential of the microbes involved in important wastewater processes such as nitrification, denitrification, enhanced biological phosphate removal (EBPR), floc formation, and solid–liquid separation. Removal of ammonium and other nitrogen species from wastewater is essential to prevent pollution and ecosystem disturbances^25,26^. Both nitrifiers and denitrifiers are required for efficient removal, by oxidizing ammonia and/or nitrite to nitrate, and then reducing nitrate to nitrogen gas. Ammonia oxidizers were represented by seven *Nitrosomonas* MAGs belonging to six different species with only one, *N. oligotropha*, representing a cultured species (Supplementary Data 3). In addition, three *Nitrospira* comammox MAGs were identified based on the presence of the genes for the ammonia monooxygenase (*amoCAB*), nitrite oxidoreductase (*nxrAB*), and hydroxylamine reductase (*hao*), and included one novel species and two *Ca*. *N. nitrosa*. The nitrifiers also included five MAGs belonging to the same species as *Nitrospira* sp. strain ND1, related to *N. defluvii* (~92% ANI)^27^, and two *Nitrotoga* MAGs belonging to a new species (94% ANI to *Candidatus* Nitrotoga sp. CP45^28^). Analysis of these new species in the 69 metagenomes revealed their presence in multiple samples, with relative abundances ranging from low up to 0.9% across the Danish WWTPs (Supplementary Fig. 5 and Supplementary Data 6). These results show that a large undescribed diversity remains hidden even within well-studied functional guilds such as nitrifiers, for which genomes will be valuable in future targeted studies.

**Fig. 4:**
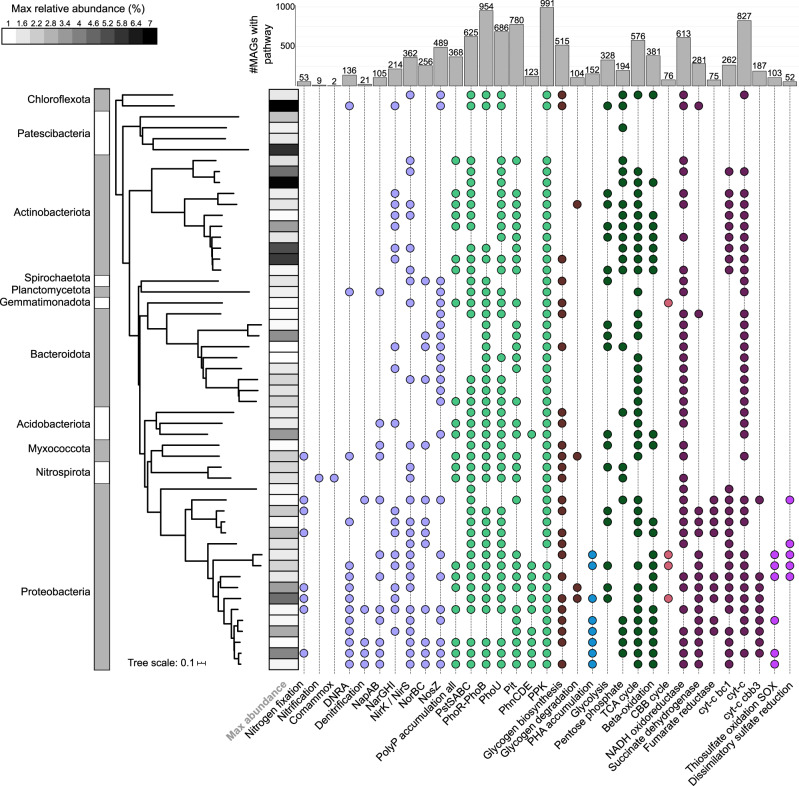
Functional profiles of the top 53 bacterial species representatives with relative abundances >1% in at least 1 sample metagenome used in this study. Pathways are considered present if 100% of the genes in the KEGG module, or custom module (Supplementary Data 12), are encoded. Heatmap strip indicates the maximum relative abundance of the population in the metagenomes. Colors are to aid visual interpretation, e.g., purple for nitrogen-related metabolisms and green for phosphate-related metabolisms. Bar chart indicates the number of MAGs encoding the pathway of interest. Supplementary Fig. 6 shows the full taxonomic string for the nodes.

Nitrate appeared to be widely used as a potential electron acceptor as many of the MAGs (214) encoded the nitrate reductase (NarGHI) (Fig. 4 and Supplementary Fig. 6). Denitrifiers encode the complete pathway for the reduction of nitrate to nitrogen gas and 21 MAGs were capable of denitrification based on the genome annotations (Supplementary Data 11). Well-known denitrifiers in AS are often found within the Gammaproteobacteria, such as *Azoarcus*, *Dechloromonas*, *Thauera*, and *Zoogloea*^29^. Similarly, most of the identified denitrifiers in the HQ MAG set belonged to the Gammaproteobacteria (15 MAGs) and included three genomes of *Zoogloea* belonging to two novel species and eight *Dechloromonas*. Interestingly, nearly half of the MAGs (489) encoded NosZ, which catalyses the reduction of N_2_O to N_2_ (Fig. 4), suggesting that non-denitrifiers are important in N_2_O reduction and potentially mitigate the release of this greenhouse gas to the environment^30^.

In contrast to denitrification, dissimilatory nitrate reduction to ammonium (DNRA) is undesired in wastewater treatment, as nitrate is converted back to ammonium rather than being removed from the system as nitrogen gas^31^. Further, DNRA microorganisms compete with denitrifiers for nitrate and can also compete for similar electron donors. Potential DNRA microorganisms processing nitrate to nitrite using NarGH and/or NapAB followed by the use of nitrite in respiration (indicated by NrfAH) were identified in 43 MAGs (Supplementary Data 11). *Geothrix* spp. enriched from AS have been found to perform DNRA when fed acetate^32^ and three *Geothrix* MAGs (two spp.) were recovered with the genomic capacity. Two *Anaeromyxobacter* MAGs also encoded the capability, which has been identified in the soil bacterium *Anaeromyxobacter dehalogenans*^33^. Additional lineages encoding DNRA genomic capabilities included two *Rubrivivax* spp. (seven MAGs, *Leptothrix* MiDAS3 tax) and a novel *Ca*. Amarolinea species (four MAGs). Potential use of the non-respiratory NirBD in DNRA was identified in numerous *Ca*. Accumulibacter, *Dechloromonas*, *Propionivibrio*, and *Ca*. Competibacter MAGs (Supplementary Data 11).

The metagenomes were derived from WWTPs performing EBPR in addition to nitrogen removal; consequently, the polyphosphate-accumulating organisms (PAOs) that are responsible for this process are of key interest. Of the well-known and confirmed PAOs, we recovered *Tetrasphaera* (14 MAGs MiDAS3 tax) and *Ca*. Accumulibacter (5 MAGs). Phosphate transporter and regulation genes (*pstABCS*, *pit*, and *phoU*) were identified in 368 MAGs in total (Supplementary Data 11 and 12, and Fig. 4). The presence of these genes does not unequivocally identify PAOs, as many microorganisms can accumulate polyphosphate without cycling it^34–36^. However, these indications of potential can be used to guide in situ tests or enrichments, to identify novel true PAOs (see below).

Glycogen-accumulating organisms (GAOs) are historically believed to compete with PAOs for substrates and have similar genome characteristics and, in some cases, similar phylogenies, to PAOs. However, GAOs do not cycle phosphate and instead use glycogen as a storage compound for use in anaerobic conditions^34^. Recent research indicates that GAOs are likely not detrimental to the activity of PAOs in full-scale systems and are abundant members of the community with many other functions^37^. Many genomes were recovered for known GAOs such as *Defluviicoccus* (three MAGs MiDAS3 tax), *Micropruina* (three MAGs), *Ca*. Competibacter (seven MAGs), *Ca*. Contendobacter (three MAGs), and *Propionivibrio* (ten MAGs)^38^, and will be valuable for confirming genus-level variation in functionality. The complete pathway for glycogen biosynthesis and degradation was encoded in 79 MAGs in total (Supplementary Data 11 and Fig. 4), reinforcing knowledge that this storage polymer is common among AS microorganisms^38^.

Many PAOs and GAOs rely on the carbon storage compound polyhydroxyalkanoates (PHAs) when conditions become anaerobic during EBPR^36^. We identified the required metabolic pathway in 152 MAGs (Fig. 4), including the experimentally validated PHA-accumulating AS populations *Dechloromonas* (eight MAGs), *Ca*. Accumulibacter (five MAGs), *Propionivibrio* (ten MAGs), and *Zoogloea* (three MAGs)^39,40^. Excluding *Austwickia* (Actinobacteriota), additional unknown but potential PHA-accumulating populations were all from the Alpha- and Gammaproteobacteria, and predominantly belonged to the genera *Rubrivivax* (13 MAGs), *Rhodoferax* (15 MAGs), and UBA1936 (10 MAGs).

Microbial morphology, such as filamentous growth, has a large impact on wastewater treatment efficiency, as specific populations are essential for flocculation, settling, and dewatering in AS, but overgrowth can lead to problems in sludge settleability (bulking) and foaming^41^. This leads to solids carryover and reduction in effluent quality, with downstream negative effects on both environmental and human ecosystem health^41^. Numerous filamentous taxa were recovered in the HQ MAG set, based on the identification of MAGs belonging to known filamentous genera^41^, including the key groups *Ca*. Microthrix (10 MAGs), *Ca*. Promineofilum (1 MAG), *Ca*. Villigracilis (14 MAGs MiDAS3 tax), *Ca*. Amarolinea (4 MAGs MiDAS3 tax), and *Leptothrix* (9 MAGs MiDAS3 tax) (Supplementary Data 3).

The recovery of 347 MQ Patescibacteria MAGs, of which 30 were circular, suggested a prevalence and likely functional importance of this group not previously recognized in AS. Saccharimonadia (formerly TM7) lineages previously described and visualized using fluorescence in situ hybridization (FISH) were consistently abundant^42,43^, but additional Patescibacteria groups were also found in high relative read abundances of up to 5.9% (Fig. 1 and Supplementary Data 6). This included two MAGs from the class Paceibacteria, belonging to different families, with 5.9% (closed CMAG EsbW_MAXAC.283) and 1.7% (single contig MQ MAG EsbW_BAT3C.204) relative abundances in the WWTP metagenome of Esbjerg W (Supplementary Note 1 and Supplementary Data 13). The highly abundant Paceibacteria were visualized with FISH, using probes developed from the recovered MAG full-length 16S rRNA genes (Supplementary Fig. 7 and Supplementary Table 1). Cells were visualized in two samples, which indicated very small populations at the limits of what could be resolved by the confocal microscopy equipment. The Patescibacteria are known to incorporate ultra-small (<0.2 μm) lineages, based on super-resolution or electron microscopy^20,44^, and we hope that others will take on the challenge of resolving this group with the necessary equipment. In Esbjerg W, where an abundance of >7% was determined in the metagenome suggesting a bloom event (Supplementary Data 3), they appeared as widespread consistent clusters, whereas in Viborg (abundance 0.7%) they appeared as isolated clusters edging the AS flocs (Supplementary Fig. 8). Due to their reduced genomes lacking many near-universal single-copy marker genes and biosynthetic capabilities, the Patescibacteria are predicted to be primarily host-dependent as either syntrophs or parasitic populations^45^. We believe this could be the case of the Paceibacteria lineage, based on the small cell size and small genome-size characteristic of Patescibacteria host-dependent cells (Supplementary Data 3). However, we were unable to determine whether the population targets specific or multiple hosts and we encourage other groups to investigate this lineage with the necessary equipment. Overall, the abundances and diversity of the Patescibacteria suggest they likely have significant involvement in AS microbial dynamics warranting further investigation.

### Linking HQ MAGs to amplicon data

Application of the MIMAG HQ benchmark would greatly benefit the validity of investigations into uncultured microorganisms through reduced chimerisms in bins, better gene syntenic information due to improved assembly contiguity, and the recovery of multicopy genes and conserved single-copy genes that are normally missing in short-read assemblies^22^. In our case, using HQ MAGs with full-length 16S rRNA genes allowed us to connect the recovered MAGs to amplicon sequencing data from the MiDAS3 project^13^, thus fulfilling the essential purpose of the MiDAS genome database: to link function to long-term structure trends and process data (Fig. 1). The 13 years of amplicon data from 20 WWTPs allowed for the identification of the consistently abundant and widespread populations, and prospective target microorganisms for closer investigation. The MAG 16S rRNA genes were mapped to the MiDAS3 full-length rRNA gene database and taxonomy, which revealed we had recovered HQ representatives for 65 of the top 100 species found in WWTP across Denmark (Supplementary Data 14)^13^. At the amplicon sequence variant (ASV) level, providing the highest level of resolution for amplicon data and a potential for species-level resolution^46^, 58 of the top 100 most abundant ASVs across Danish WWTPs (averaged relative abundances across MiDAS3) were recovered in an HQ MAG (Supplementary Data 15). These recovered populations are not only abundant in Danish systems but likely represent important populations in nutrient-removal plants across the world^13,46^.

**Fig. 5:**
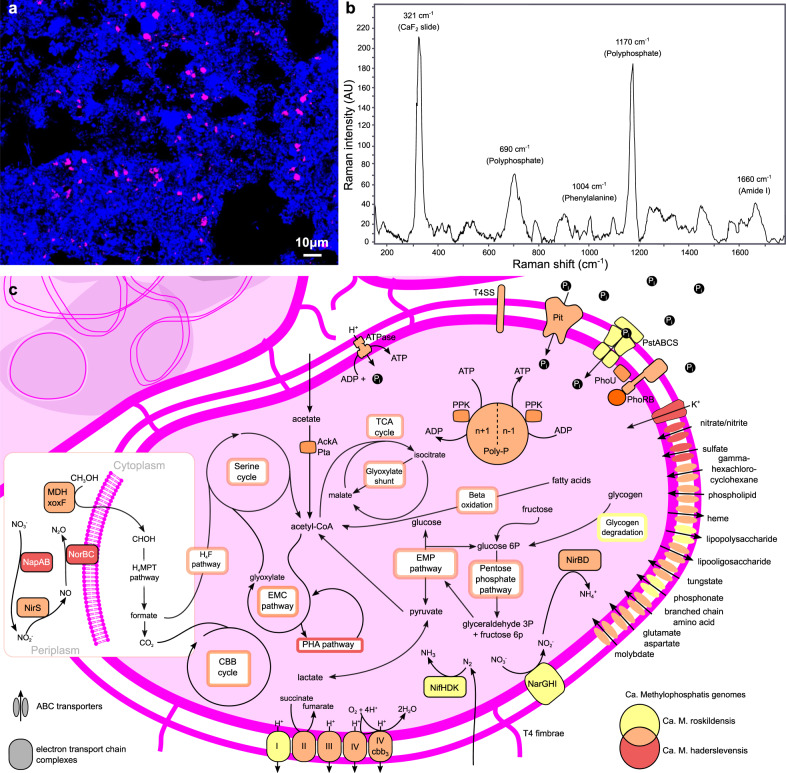
Overview of *Ca*. Methylophosphatis based on FISH, Raman microspectroscopy, and metabolic reconstruction. **a** FISH micrograph of *Ca*. Methylophosphatis, targeted by the genus-specific probe g190_1276 (Cy3-labeled) in a full-scale activated sludge sample from Bjergmarken WWTP (2018-08-29). Two samples were examined in total and multiple images were recorded for each sample. Source data are provided as a Source Data file. Target cells appear magenta, whereas all other bacterial cells appear blue. Scale bar represents 10 μm. **b** Raman spectrum of *Ca*. Methylophosphatis (average of 100 FISH-defined cells) showing the presence of the signature peaks for polyphosphate (690 and 1170 cm^−1^). Peaks for phenylalanine (1004 cm^−1^) and amide I linkages of proteins (1450 cm^−1^) are specific markers for biological material. AU, arbitrary units. **c** Metabolic reconstruction of the *Ca*. Methylophosphatis MAGs. Colors represent the species or combination of species (Venn diagram) that encode the potential for the enzyme or cycle. Abbreviations: EMC, ethylmalonyl-CoA pathway; EMP, Embden–Meyerhof–Parnas pathway (glycolysis); CBB, Calvin–Benson–Bassham cycle; H_4_MPT, tetrahydromethanopterin pathway; H_4_F, tetrahydrofolate pathway; TCA, tricarboxylic acid cycle; PHA, polyhydroxyalkanoate pathway, nitrogenase (NifHDK); CH_3_OH, methanol, methanol dehydrogenase (MDH-xoxF); I, complex I NADH dehydrogenase; II, complex II succinate dehydrogenase; III, complex III cytochrome bc1; IV, cytochrome *c* oxidase; IV cbb3, complex IV cytochrome cbb3 oxidase, inorganic phosphate transporter family (Pit), inorganic phosphate ABC transporter (PstABCS), two component system for phosphate regulation (PhoRB), phosphate transport system accessory protein (PhoU); Poly-P, polyphosphate, type IV secretion system (T4SS), type IV fimbriae (T4 fimbriae), nitrate reductase respiratory (NarGHI), periplasmic nitrate reductase (NapAB), nitrite reductase (NirS), nitric oxide reductase (NorBC), acetate kinase (AckA), and phosphotransacetylase (Pta).

The 16S rRNA gene amplicon data was linked to the MAG metabolic annotations, to investigate novel and consistently abundant populations with metabolisms relevant to resource recovery and bioremediation, such as the polyphosphate, PHA, glycogen, and denitrification metabolisms described above. Based on the MAG 16S rRNA gene sequences and previous efforts to recover full-length 16S rRNA gene sequences from AS^13,46^, FISH probes were designed to target a potential polyphosphate-accumulating population belonging to a novel genus, midas_g_190, most closely related to *Methyloversatilis* (<78% ANI) (Supplementary Table 1 and Supplementary Note 2). Phylogenetic analysis of these sequences revealed an evident separation into three species, providing an overview of the novel genus (Supplementary Fig. 9). Application of the designed probes revealed 0.8–1 × 0.5–0.6 μm rod-shaped cells that were often arranged in microcolonies inside the flocs, or sometimes attached to filaments (Fig. 5a). The relative abundances determined by amplicon sequencing were in the same range as quantitative FISH (Supplementary Table 2). Two species-level MAGs were recovered from within this genus, with four MAGs belonging to midas_s_484 (completeness: 91.2–96.8%; contamination: 2–4.2%) and one MAG belonging to midas_s_514 (completeness: 91.2%; contamination: 2.4%). Using FISH-Raman microspectroscopy for the analysis of intracellular storage polymers, all FISH-defined cells belonging to the genus were experimentally determined to store polyphosphate at levels (i.e., amounts) similar to known PAOs such as *Ca*. Accumulibacter (see “Methods”), confirming the metabolism observed in the genomes (Fig. 5b, c, and Supplementary Data 16), and suggesting this population may have an important role in phosphate recovery in the AS system. No Raman peaks were found for the other intracellular storage compounds glycogen and PHA. Due to genomic and experimental evidence for phosphate accumulation, and the genomic potential of methylotrophy combined with relatedness to a characterized methylotrophic genus, we propose the names *Ca*. Methylophosphatis haderslevensis (midas_s_514) and roskildensis (midas_s_484) for the two species-level populations.

## Conclusion

Here we present a methodology for the high-throughput production of HQ MAGs and its application to investigate complex microbial communities, focusing on the AS system. This approach enabled amplicon and full-length 16S rRNA gene data to be linked to the functional potential of the entire HQ MAGs. We show that producing HQ MAGs from complex environments is increasingly an affordable and feasible undertaking (in our case ~$12 USD per MQ-HQ MAG). Raising MAG standards facilitates the identification and experimental confirmation of key functional microbes, demonstrated by our characterization of the polyphosphate-accumulating novel genus *Ca*. Methylophosphatis. Furthermore, raising standards will also aid exploratory studies by preventing contamination of public repositories with low-quality MAGs^4^. We expect that the rate of improvement in DNA extraction methods and long-read sequencing technologies indicates that thousands of HQ MAGs and CMAGs are on the horizon.

### Etymology

Description of “*Candidatus* Methylophosphatis” gen. nov. (midas_g_190): “*Candidatus* Methylophosphatis” [Me.thy.lo.phos.pha’tis. N. L. n. methylum (from French *méthyle*, back-formation from French *méthylène*, coined from Gr. n. *methu*, wine and Gr. n. *hulê*, wood), the methyl group; N.L. pref. *methylo*-, pertaining to the methyl radical; *phosphatis* from L. nm. phosphas, phosphate: a likely methylotroph that can accumulate polyphosphate].

Description of “*Candidatus* Methylophosphatis roskildensis” sp. nov. (midas_s_484): “*Candidatus* Methlylophosphatis roskildensis” [ros.kil.den’sis. N. L. fem. adj. roskildensis pertaining to the city of Roskilde, the city in which the Bjergmarken WWTP is situated and where the sample with the most abundant population was obtained].

Description of “*Candidatus* Methylophosphatis haderslevensis” sp. nov. (midas_s_514): “*Candidatus* Methylophosphatis haderslevensis” (midas_s_514) [ha.der.sle.ven’sis. N. L. fem. adj. haderslevensis pertaining to the city of Haderslev, where the sample was obtained from which the MAG was produced].

## Methods

### Sample processing and DNA extractions

Fresh AS samples were received from 23 Danish WWTP in August and September 2018. Samples were aliquoted into 1.6 mL replicates and frozen at −80 °C prior to processing. For extraction, each 1.6 mL tube was decanted into an empty PowerSoil 2 mL tube. The sample was then spun at 10,000 × *g* for 5 min and most supernatant was discarded. PowerSoil beads were transferred back to the tube with the pelleted AS and the remaining steps followed the DNeasy PowerSoil Kit (Qiagen, Germany) protocol, except vortexing steps were substituted with gentle tube inversion (×10) and the DNA was eluted from the spin column using 60 µl of solution C6. Complementary DNA for differential coverage binning was selected from 2016 and 2017 for the same plants. This DNA was extracted previously using the FastSpin DNA kit for soil (MP Biomedicals) as described in ref. ^13^. Briefly, extraction followed the manufacturer’s protocol, except for the increase of bead beating to 4 × 40 s at 6 m/s using the FastPrep FP120 (MP Biomedicals). The WWTP Viby was new to the MiDAS program in 2018, so DNA extracted using FastSpin from February and May of 2018 was selected for differential coverage. DNA was temporarily stored in a −20 °C freezer until size selection. Size selection of the DNA followed the adapted Oxford Nanopore community SPRI protocol originally adapted from Schalamun and Schwessinger^47^. DNA concentration and quality was checked using the Agilent TapeStation genomic DNA screen tapes, Qubit 2.0 fluorometer (Thermo Fisher Scientific, MA, USA), and Nanodrop ND1000 (Thermo Fisher Scientific, MA, USA). After size selection, DNA was stored in TE buffer (pH 8).

### Library preparation and sequencing

Library preparation for Oxford Nanopore sequencing used the one-pot native barcoding protocol^48^ with the EXP-NBD104 native barcodes, followed by Oxford Nanopore 1D Genomic DNA by ligation (SQK-LSK109) (PromethION) according to the manufacturer’s instructions. Samples were barcoded and then pooled in groups of three samples at equimolar concentrations. Pooled samples were run on the Oxford Nanopore PromethION R9 FLO-PRO002 flow cells. If samples did not reach the desired output of at least 20 Gbp of data, they were re-run and the data pooled. Eleven flow cells were used in total and each flow cell was run within 8 weeks of delivery. Flow cells were run for 48 h.

For Illumina sequencing, genomic DNA was quantified with Qubit 2.0 DNA HS Assay and quality assessed by agarose gel imaging. Library preparation and sequencing of the Illumina data were conducted through Genohub (Austin, TX) and Admera Health (South Plainfield, NJ). Libraries were constructed using a PCR-free workflow following the manufacturer’s protocol of NEBNext® Ultra™ II DNA Library Prep Kit for Illumina targeting an insert size of 300–400 bp and run on an Illumina Hiseq X generating 922.5 Gbp of 2 × 150 bp PE data. Library QC was performed with Tapestation D1000 Assay (Agilent Technologies, CA, USA) and quantified using qPCR with QuantStudio ® 5 System (Applied Biosystems, USA) (Supplementary Data 1).

### Basecalling and data QC

The 17TB of raw Nanopore fast5 data was basecalled using Guppy v2.2.3 for PromethION and the dna_r9.4.1_450bps_flipflop_prom.cfg model from Oxford Nanopore. 1081 Gbp of data were produced in total from the flow cells after basecalling, and after demultiplexing the yield ranged from 21 to 59 Gbp per sample (Supplementary Data 1 and 2). The data were explored using MinionQC v1.4.0^49^. Samples were demultiplexed, and barcodes and adapter sequences trimmed using qcat v1.0.1 (https://github.com/nanoporetech/qcat), using the following flags -b -k NBD103/NBD104 --trim --detect-middle. Following demultiplexing, the Nanopore reads were further processed using Filtlong v0.2.0 (https://github.com/rrwick/Filtlong), to remove reads <4000 bp using –min_length 4000 and to remove low-quality reads with <80% base call accuracy using –min_mean_q 80. Porechop v0.2.3 (https://github.com/rrwick/Porechop) was used to check the reads for residual barcodes and adapters using default settings and the flag –min_split_read_size 4000, and this was followed by a final round of Filtlong using the described parameters.

The 0.92 Tbp of Illumina data was quality checked using FastQC v0.11.7^50^ and MultiQC v1.7^51^. Cutadapt v1.16^52^ removed the sequencing adapters, reads < 100 bp, reads with a qscore <20, trimmed ambiguous bases (N) from the start and end, and removed reads with ambiguous bases using the parameters -m 100 -q 20 --max-n=COUNT 0 --trim-n.

### Metagenome assembly and polishing

Long-read assembly and polishing followed the methodology below and used GNU parallel v20190122^53^ extensively. The quality-processed Nanopore reads were assembled using CANU v1.8^54^, using the following parameters: corMinCoverage=0 corOutCoverage=all corMhapSensitivity=high correctedErrorRate=0.105 genomeSize=5m corMaxEvidenceCoverageLocal=10 corMaxEvidenceCoverageGlobal=10 oeaMemory=32 redMemory=32 batMemory=200. If the assembly failed due to set memory limitations, the genome size was increased to -gs 50000000 and the script restarted from the fail point, as suggested by the developers. Once the assemblies were produced, MUMmer v3.2.3^55^ nucmer was used on the contigs CANU labeled as circular (suggestCircular=yes) to show overlaps, so that the duplicated sequence could be removed using R v3.5.0^56^ and unix cut prior to assembly polishing. Contigs < 4000 bp were removed using seqtk v1.3-r106 (https://github.com/lh3/seqtk) seq. Nanopore assembly polishing was accomplished using Racon v1.3.3 (https://github.com/lbcb-sci/Racon) with the argument --include-unpolished for initial correction based on the mapping of the quality processed nanopore reads using minimap2 v2.16^57^ -x map-ont. This was followed by two rounds of medaka v0.6.5 (https://github.com/nanoporetech/medaka), comprising mini_align, medaka consensus using the r941_flip213 model, and medaka stitch commands. For medaka consensus, the assemblies were split into first 10 (for medaka round 1) and then 20 (for medaka round 2) different files with the contig headers space separated and provided to the --region flag. This was done at the developer’s suggestion, to improve processing time. Following the second round of medaka, minimap2 was used to map the quality-processed Illumina reads to the medaka polished assembly and Racon --include-unpolished was used again with these reads for a final round of error correction. Samtools v1.9^58^ was used as a dependency for this pipeline.

### MAG binning and dereplication

Binning followed the pipeline mmlong v0.1.2 hybrid-metaflow after the assembly step (https://github.com/SorenKarst/mmlong). Briefly, Nanopore and Illumina reads were mapped to the polished Nanopore assembly using minimap2 v2.15. Automatic binning was conducted using MetaBAT2 v2.12.1^59^ and MaxBin v2.2.7^60^. Metagenome contigs were translated into proteins using FragGeneScan v1.31^61^, annotated taxonomically using Kaiju v1.6.0^62^ against the proGenomes database (2017-05-16), 16S rRNA genes were identified with barrnap v0.9 (https://github.com/tseemann/barrnap), and then classified with MOTHUR v2.7.14 classify.seqs against the SILVA v132 seed database^63^. Binning was conducted using two coverage approaches for each of the two binning tools, first using the differential coverage information from only the same plant as the assembly (i.e., the corresponding three Illumina metagenomes 2016, 2017, and 2018) or differential coverage information from all of the Illumina metagenomes (69 in total). DASTool v1.1.1^64^ --search_engine diamond was used to dereplicate and select for the best representative bin from the four binning iterations for each of the 23 metagenomes.

The dereplicated bins were then checked for completeness and contamination using CheckM --lineage_wf^65^ v1.0.11, resulting in 3733 MQ to HQ MAGs. Circular genomes were identified by linking the contig names back to the suggestCircular=yes CANU designations. Circular contigs > 700 kbp were identified as likely circular chromosomes. Ultra-small genomes with a circular chromosome were included in the HQ set, despite not full-filling the completeness cut-off of >90%.

Five MAGs failed the contamination threshold and were manually examined using mmgenome2 v2.0.7 (https://github.com/KasperSkytte/mmgenome2), four were circular, and one was a MAG of interest (*Nitrospira*). Likely contaminating contigs were removed and quality was re-checked with CheckM. These manually improved MAGs are identified by “v2” in the MAG ID in the Supplementary Tables. Of 43 MAGs containing a circular contig of >700,000 kb, 29 circular MAGs were manually checked and additional non-circular extraneous contigs were removed. These are identified by “cln” in the MAG ID. Three MAGs that contained circular contigs but also large additional linear contigs encoding single-copy marker genes were potentially multichromosomal and were removed from the CMAG designations.

dRep v2.3.2^66^ -comp 50 -con 10 dereplicated the MAGs at 99% ANI clustering to indicate the number of distinct lineages and overlap of likely strains between WWTPs, and at 95% ANI to indicate the number of distinct species.

### Reassembly of 17 Patescibacteria additional CMAGs

Although 13 circular Patescibacteria MAGs were initially recovered in the automated binning run with CANU, there were hundreds of additional MQ Patescibacteria MAGs (334) and many had high relative abundances in the metagenomes (e.g., EsbW_18-Q3-R4-48_MAXAC.283, Supplementary Data 3). MQ Patescibacteria MAGs with >30% of their total base pairs in the longest contig were selected for reassembly with Flye v2.6^67^ and Unicycler v0.4.6^68^. First, the Nanopore and Illumina reads that had mapped to bin contigs in the original CANU assemblies were extracted using samtools. These were used as input to Unicycler (unicycler -1 -2 -l --no_correct --min_kmer_frac 0.3 --kmer_count 5 --no_pilon --keep 3 --mode bold --min_fasta_length 1000) and Flye (--nano-raw --genome-size 1m). The Flye and Unicycler assembly graphs were checked in Bandage v0.8.1^69^, to determine which assemblies were now circular. Overall, 11 additional circular Patescibacteria MAGs were recovered with Flye and 6 additional were recovered using Unicycler. These MAGs are identified by “fly” or “uni” in the MAG IDs. The circular contigs from the Flye assemblies were further polished with minimap2, Racon with Nanopore reads, medaka, and Racon with Illumina reads following the method used for the CANU assembly above, except medaka_consensus was used instead of the mini_align, medaka consensus, and medaka stitch substeps. The Flye MAGs were checked for circularity by ensuring reads mapped across the contig start and end based on manual inspection in Tablet^70^. EsbW_18-Q3-R4-48_MAXAC.283 was further refined based on the mappings, to include split tandem repeats flanking the viral sequence, which were present in the mapped reads but had been trimmed by the assembly.

Polymorphic rates for all HQ MAGs were determined using CMSeq as described in Pasolli et al.^3^ (https://bitbucket.org/CibioCM/cmseq/), using the poly.py and polymut.py scripts, and --mincov 10 --minqual 30 --dominant_frq_thrsh 0.8 (Supplementary Data 3).

### MAG taxonomy and phylogeny

Genome taxonomy was determined using GTDB-Tk v1.0.2^71^ and the refseq release 89 (2019-06-19) database, and the dependencies pplacer v1.1^72^, FastANI v1.2^73^, Prodigal v2.6.2^74^, FastTree 2 v1.2^75^, and HMMER v3.1b2^76^ (Supplementary Data 3). Both MQ (MAGs with >50% completeness, <10% contamination) and HQ MAGs were classified. ARB v6.0.3^77^ and IToL v5.5^78^ were used for visualizing and refining the tree created by GTDB-Tk, and for illustrating the MAG phyla in Fig. 2. Pavian^79^ was used to create the Fig. 3a Sankey and is based on the taxonomy assigned by GTDB-Tk.

### Genome annotations and 16S rRNA gene mapping to MiDAS3

Prokka v1.14 --meta --kingdom Bacteria or Archaea^80^ and Infernal v1.1.2^81^ (arguments: cmscan --cut_ga --rfam --nohmmonly --fmt 2) were run on the MAGs > 90% complete and <5% contamination, and the reduced genome circular MAGs to identify the 16S, 23S, 5S rRNA, and tRNA genes. Only the MAGs with full-length rRNA genes and >18 tRNA genes determined with either Prokka and/or Infernal were perceived as HQ and were selected for further analyses. Partial matches in Prokka or Infernal were discarded. 16S rRNA gene sequences were extracted using BEDTools v2.27^82^ or Fxtract v2.3 (https://github.com/ctSkennerton/fxtract). MAG 16S sequences were mapped against the SILVA v138 nr99 database^83^, and MiDAS full-length ASV (FL-ASV) and ASV databases using USEARCH v11^84^, first orientating the sequences against SILVA with usearch11 -orient, then using -usearch_global -top_hit_only -strand plus -id 0.99 (ASV) -id 0.7 (FL-ASV and SILVA) -maxaccepts 0 -maxrejects 0 -blast6out. The MAG ASV and FL-ASV designations were matched to those in the MiDAS3 database, to determine how many of the top 100 species or ASVs had been recovered in an HQ MAG^13^. Primer mismatches to the 27f and 534r primers for the V1-3 regions of the 16S rRNA genes of the MAGs were determined using PrimerProspector v1.0.1^85^ analyze_primers.py and taxa_coverage.py (Supplementary Data 3). MAG genome size, longest contig length, and average contig length were calculated using the esl-seqstat program downloaded alongside Infernal (Supplementary Data 3).

EnrichM v0.5.0 (https://github.com/geronimp/enrichM) annotate using Diamond v0.9.22^86^ blasted the MAG proteins against the EnrichM v10 database, incorporating a KO-annotated uniref100 database. EnrichM “classify” was used to reveal the complete KEGG Orthology (KO) modules present in the MAGs. The modules searched for included custom module files from Woodcroft et al.^8^ and this study (Supplementary Data 12). These data were used for the construction of the metabolism Fig. 4. The GTDB taxonomy is used for the description of the functional guilds and taxonomy throughout the study, except where the MiDAS3 taxonomy is explicitly stated in the text.

### Mean coverage, relative abundances, and recovery success of MAGs

Depth of coverage (mean) based on the Illumina data and Nanopore data was calculated using CoverM v0.3.2 (https://github.com/wwood/CoverM) and the filtered bam files created during the mmlong process described above, where the metagenome Illumina and Nanopore reads were mapped directly to the corresponding metagenome assembly (Supplementary Data 3). The following arguments were used: coverm genome -m mean --min-read-aligned-percent 0 --min-read-percent-identity 0 --min-covered-fraction 0. Relative abundances of the MAG species representatives in the metagenomes were calculated by mapping the Illumina data for each of the 69 metagenomes to a concatenated fasta file of the 581 bins using default CoverM settings, except the following arguments: coverm genome -m relative abundance --min-read-aligned-percent 0.75 --min-read-percent-identity 0.95 --min-covered-fraction 0. Stringent identity and alignment cutoffs were used to minimize spurious mappings falsely inflating abundances.

The proportion of the metagenome community recovered in the assembly or the HQ MAG set was investigated using SingleM v0.12.1 (https://github.com/wwood/singlem). SingleM identifies single-copy marker genes of 14 ribosomal proteins in short-read data, assemblies, and bins, and avoids the complications of MAG recovery estimates based on multicopy 16S rRNA genes (https://github.com/wwood/singlem). SingleM pipe was run on the individual metagenomes, assemblies, and HQ MAGs. SingleM summarize was then run on the SingleM pipe MAG operational taxonomic unit (OTU) files to concatenate them into one large table for singlem appraise. This table of OTUs, representing 14 single-copy marker genes, could then be compared to the metagenomes with their corresponding assemblies using singlem appraise. This allowed us to determine the percentage of the metagenome community recovered at each step. For the genus-level recovery estimates, the flags --imperfect --sequence_identity 0.89 were used to cluster the OTUs at 89% ANI, or roughly the genus level^8^.

Unbinned populations were also identified using the SingleM data, specifically the OTUs that were abundant in the metagenomes but not matched to the HQ MAG set. Here, “singlem pipe” was run with a stringent evalue of “1e-20” on the HQ MAGs to avoid spurious hits to homologous regions, and “singlem appraise” with the flag --output_unaccounted_for_otu_table was used to produce an output table of the unbinned hits (present in the metagenome but not the HQ MAGs). This table was transformed into biom format using “singlem summarise” --biom_prefix and these tables were imported into R v3.5.2 using the ampvis2 v2.5.8. R package (https://github.com/MadsAlbertsen/ampvis2). Relative abundance heatmaps of the unbinned populations (Supplementary Figs. 2–4) were produced with these data. These data were also used to examine the envOPS12 populations that were not successfully recovered with “singlem query.” The unbinned marker gene sequences of envOPS12 were used as input to query the MQ and low-quality bins of EsbE and EsbW metagenomes.

### Phylogenetic analysis, probe design, and FISH of Paceibacteria and *Ca*. Methylophosphatis

Phylogenetic analysis of 16S rRNA gene sequences and FISH probe design were performed using ARB v. 6.0.6^77^. Analysis of 16S rRNA gene sequences from the Paceibacteria MAGs was conducted by aligning sequences with MAFFT v7.402^87^, trimming with TrimAL v1.4.rev15^88^ to remove bases with <90% coverage but conserving 60% of the original alignment, and creating a phylogenetic tree in IQ-TREE v1.5.6 ^89^ using RAxML GTR algorithm with 100 bootstraps. Two FISH probes, Pac_113 and Pac_683, were used to investigate the lineages (Supplementary Table 1). For *Ca*. Methylophosphatis, a phylogenetic tree was calculated based on the aligned 12 FL-ASVs from the genus midas_g_190 and the 16S retrieved from the MAGs, using the PhyML maximum likelihood method and a 1000-replicate bootstrap analysis. Unlabeled competitor probes were designed for single-base mismatched non-target sequences for *Ca*. Methylophosphatis (Supplementary Table 1). Both sets of probes were validated in silico with mathFISH^90^, to test the hybridization efficiency of target and non-target sequences (Supplementary Figs. 10 and 11). The number of non-target sequences with 0, 1, and 2 mismatches was assessed using the probe match function in ARB. All probes were purchased from Biomers (Biomers.net, Ulm, Germany) and were labeled with ATTO 532 or ATTO 594 fluorochromes (Paceibacteria), or labeled with indocarbocyanine (Cy3) or indodicarbocyanine (Cy5) fluorochromes (*Ca*. Methylophosphatis).

For *Ca*. Methylophosphatis, optimal hybridization conditions for the FISH probes were determined based on the formamide dissociation curves generated after hybridization at different formamide concentrations over a range of 0–70% (v/v) with 5% increments. Relative fluorescence intensities of 50 cells were measured with the ImageJ software (National Institutes of Health, Maryland, USA) and the calculated average values were compared for selection of the optimal formamide concentration. As a pure culture was not available, the probes were optimized using AS biomass with a high abundance of the target organism predicted by amplicon sequencing. An unlabeled competitor probe was included in every analysis performed and their use is recommended in future studies. Details about the optimal formamide concentration used for each probe are given in Supplementary Table 1.

Fresh biomass samples from full-scale AS WWTPs were fixed with 4% paraformaldehyde (final concentration) for 3 h at 4 °C and washed three times with 1 mL of sterile filtered tap water, and stored in the freezer (−20 °C) until needed. FISH was performed as described by refs. ^91,92^. Briefly, 8 ml of hybridization solution containing 30 ng of FISH probe was applied to the sample immobilized and dehydrated on a slide, and incubated for 3 h at 46 °C in a humid chamber. Afterwards, the slides were first washed with 2 ml of washing solution and then immersed in 50 ml of pre-warmed (48 °C) washing solution and incubated for 15 min. The slides were rinsed briefly with cold distilled water and air-dried. The EUBmix probe set was used to cover all bacteria^93,94^ and the nonsense NON-EUB probe was applied as negative control for sequence independent probe binding^95^.

Microscopic analysis was performed with either an Axioskop epifluorescence microscope (Carl Zeiss, Germany), equipped with a Leica DFC7000 T CCD camera, or a white light laser confocal microscope (Leica TCS SP8 X) (Leica Microsystems, Wetzlar, Germany). For *Ca*. Methylophosphatis, images taken at the confocal laser scanning microscope and the software ImageJ were used to measure cell size. Only free single cells (few but present) were used for the measurements. The resulting lengths and diameters of the rod-shaped cells of *Ca*. Methylophosphatis were the average of 100 cells measured. Quantitative FISH (qFISH) biovolume fractions of individual taxa (*Ca*. Methylophosphatis) were calculated as a percentage area of the total biovolume, hybridizing the EUBmix probes, which also hybridizes with the specific probe. qFISH analyses were based on 30 randomly chosen fields of view taken at ×63 magnification using the Daime image analysis software^96^.

### Raman microspectroscopy of *Ca*. Methylophosphatis (midas_g_190)

Raman microspectroscopy was applied in combination with FISH, as previously described^97^, to detect the presence of intracellular storage polymers. Briefly, FISH was conducted on optically polished CaF_2_ Raman windows (Crystran, UK), which give a single-sharp Raman marker at 321 cm^−1^ that serves as an internal reference point in every spectrum. The genus-specific probe for *Ca*. Methylophosphatis (midas_g_190, Supplementary Table 2) was used to locate 100 target cells for Raman analysis. Two spectra were acquired per cell. After bleaching the Cy3 fluorophore with the Raman laser, spectra from single cells were obtained using a Horiba LabRam HR 800 Evolution (Jobin Yvon, France) equipped with a Torus MPC 3000 (UK) 532 nm 341 mW solid-state semiconductor laser. The Raman spectrometer was calibrated prior to obtaining all measurements to the first-order Raman signal of Silicon, occurring at 520.7 cm^−1^. The incident laser power density on the sample was attenuated down to 2.1 mW μm^−2^ using a set of neutral density filters. The Raman system is equipped with an in-built Olympus (model BX-41) fluorescence microscope. A ×50, 0.75 numerical aperture dry objective (Olympus M Plan Achromat- Japan), with a working distance of 0.38 mm, was used throughout the work. A diffraction grating of 600 mm/groove was used and the Raman spectra collected spanned the wavenumber region of 200–1800 cm^−1^. The slit width of the Raman spectrometer and the confocal pinhole diameter were set to 100 and 72 μm, respectively. Raman spectrometer operation and subsequent processing of spectra were conducted using LabSpec v6.4 software (Horiba Scientific, France). All spectra were baseline corrected using a sixth-order polynomial fit. Signature peaks for storage polymers in known PAO cells were described in Fernando et al.^97^ and were used as comparison for identification of the peaks in *Ca*. Methylophosphatis cells. Briefly, signature peaks for polyphosphate have been detected at 690 and 1170 cm^−1^, for glycogen at 480 and 1765 cm^−1^ for PHA.

### Reporting summary

Further information on research design is available in the Nature Research Reporting Summary linked to this article.

## Supplementary information

Supplementary Information

Description of Additional Supplementary Files

Supplementary Data 1

Supplementary Data 2

Supplementary Data 3

Supplementary Data 4

Supplementary Data 5

Supplementary Data 6

Supplementary Data 7

Supplementary Data 8

Supplementary Data 9

Supplementary Data 10

Supplementary Data 11

Supplementary Data 12

Supplementary Data 13

Supplementary Data 14

Supplementary Data 15

Supplementary Data 16

Reporting Summary

## Data Availability

Data generated and used in this study, Illumina and Oxford Nanopore metagenomes and HQ MAGs, are deposited in the NCBI SRA and GenBank databases under the bioproject accession number PRJNA629478. Accession numbers for the MAGs are provided in Supplementary Data 3. Both MQ and HQ MAGs have been deposited in Figshare, to enable bulk download under DOI 10.6084/m9.figshare.c.5277035^98^. Source Data for the FISH images (raw TIFF format) are also available in the Figshare collection^98^. Data yield and MAG statistics are presented in the Supplementary Data Files. The EnrichM v10 database, including the KO-annotated uniref100 database, is found at https://data.ace.uq.edu.au/public/enrichm/. GTDBTk Refseq release 89 database is found at https://data.ace.uq.edu.au/public/gtdbtk/release_89/. The Kaiju proGenomes database is found at http://kaiju.binf.ku.dk/server. The MiDAS3 database is found at https://www.midasfieldguide.org/guide/downloads. SILVA v132 and v138 are found at https://www.arb-silva.de/download/archive/.

## Code availability

The methods above indicate the source and versions of the programs and code used for the analyses.
