# Supplementary material for: Connecting structure to function with the recovery of over 1000 high-quality metagenome-assembled genomes from activated sludge using long-read sequencing
Source: Nat Commun. 2021 Mar 31;12:2009. doi: 10.1038/s41467-021-22203-2 (PMC8012365; doi:10.1038/s41467-021-22203-2)
Supplement: Supplementary file 1 — Supplementary Information [file 41467_2021_22203_MOESM1_ESM.pdf]

Supplementary Information

Connecting structure to function with the recovery of over 1000 high-quality metagenome-assembled genomes from activated sludge using long-read sequencing

Caitlin M Singleton[1], Francesca Petriglieri[1], Jannie M Kristensen[1], Rasmus H Kirkegaard[1], Thomas Y Michaelsen[1], Martin H Andersen[1], Zivile Kondrotaite[1], Søren M Karst[1], Morten S Dueholm[1], Per H Nielsen[1]*, Mads Albertsen[1]*

Supplementary Figures

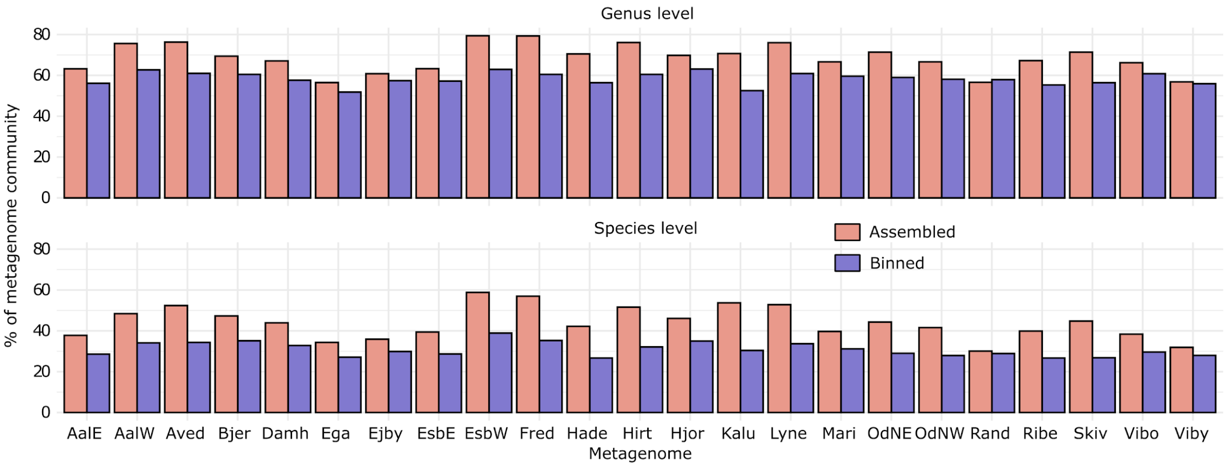

Supplementary Fig. 1: **Results of the single copy ribosomal protein analysis from SingleM, showing the proportion of the metagenome populations that were successfully assembled and binned per WWTP**. Abundance is determined from the single copy ribosomal protein gene sequences, as taxonomic units, using the information in Supplementary Data 4. n = 1 biologically independent samples. Species level and genus level recovery are shown. Orange indicates the proportion of the metagenome represented by the assembly. Purple indicates the proportion of the metagenome represented in the binned HQ MAG set.

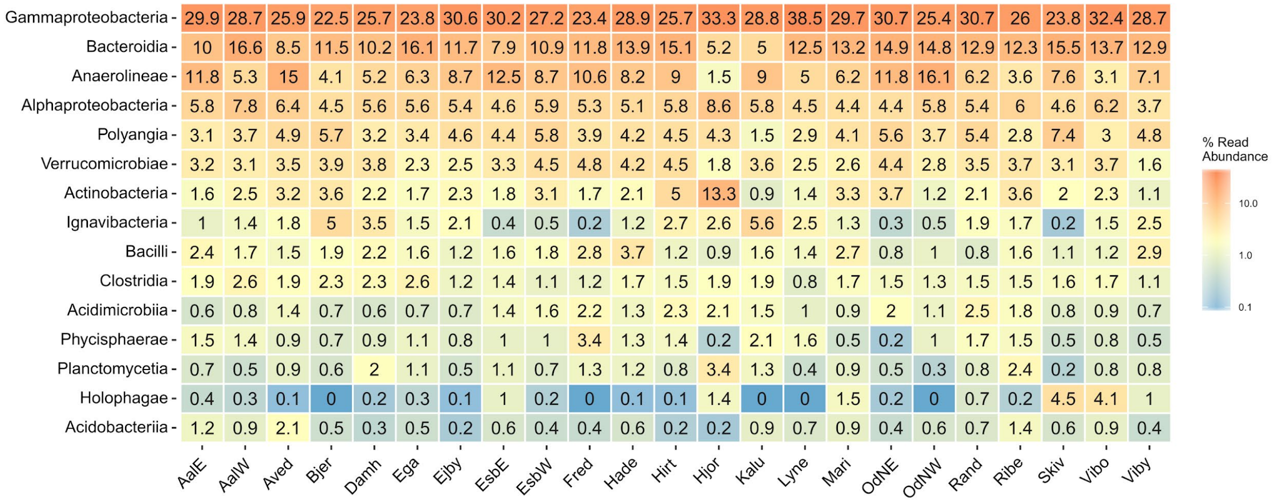

Supplementary Fig. 2: **SingleM unbinned OTUs distribution heatmap showing the top 15 classes for the marker gene *rplE* for ribosomal protein L5**. Abundance is relative to the total unbinned marker gene sequences.

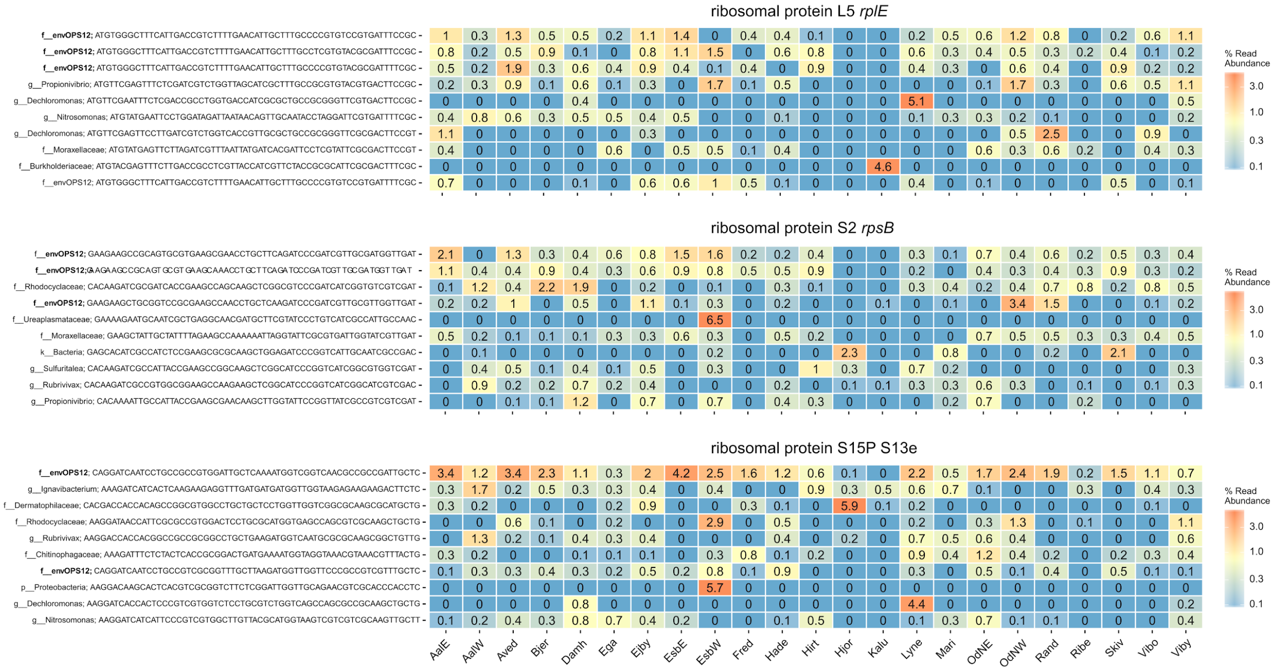

Supplementary Fig. 3: **SingleM unbinned taxonomic units (representing discrete populations) distribution heatmap of the top 10 populations for three ribosomal protein marker genes**. The envOPS12 populations are bolded. Abundance is relative to the total unbinned marker gene sequences.

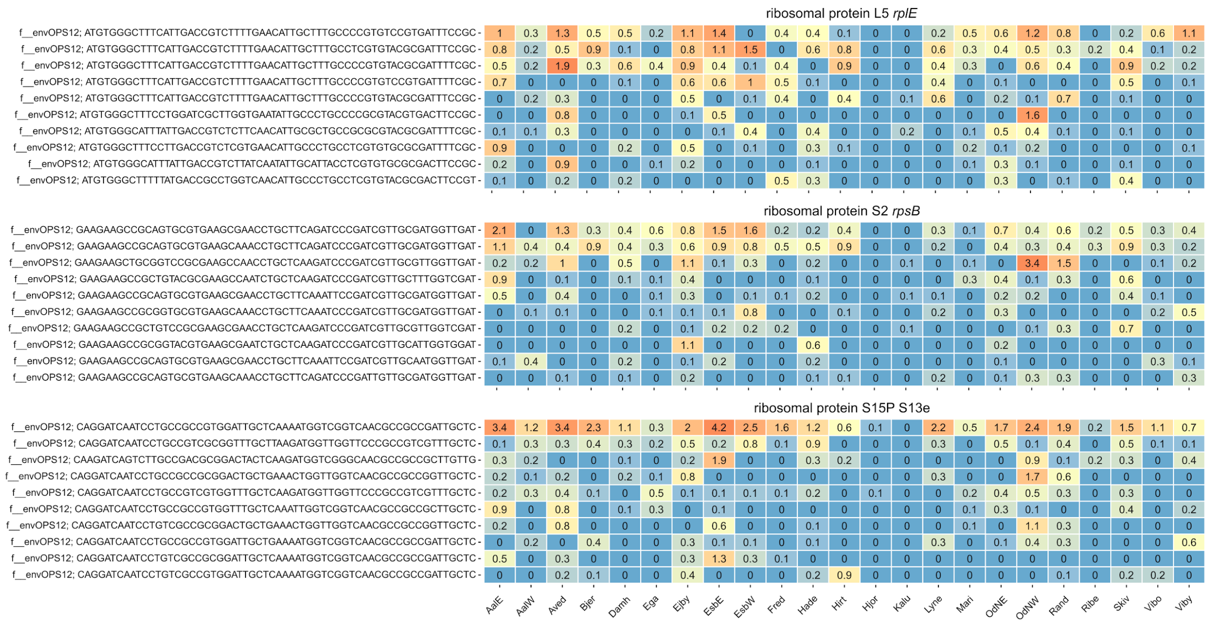

Supplementary Fig. 4: **SingleM unbinned envOPS12 taxonomic unit distribution heatmap of the top 10 populations for three ribosomal protein marker genes**. Abundance is relative to the total unbinned marker gene sequences.

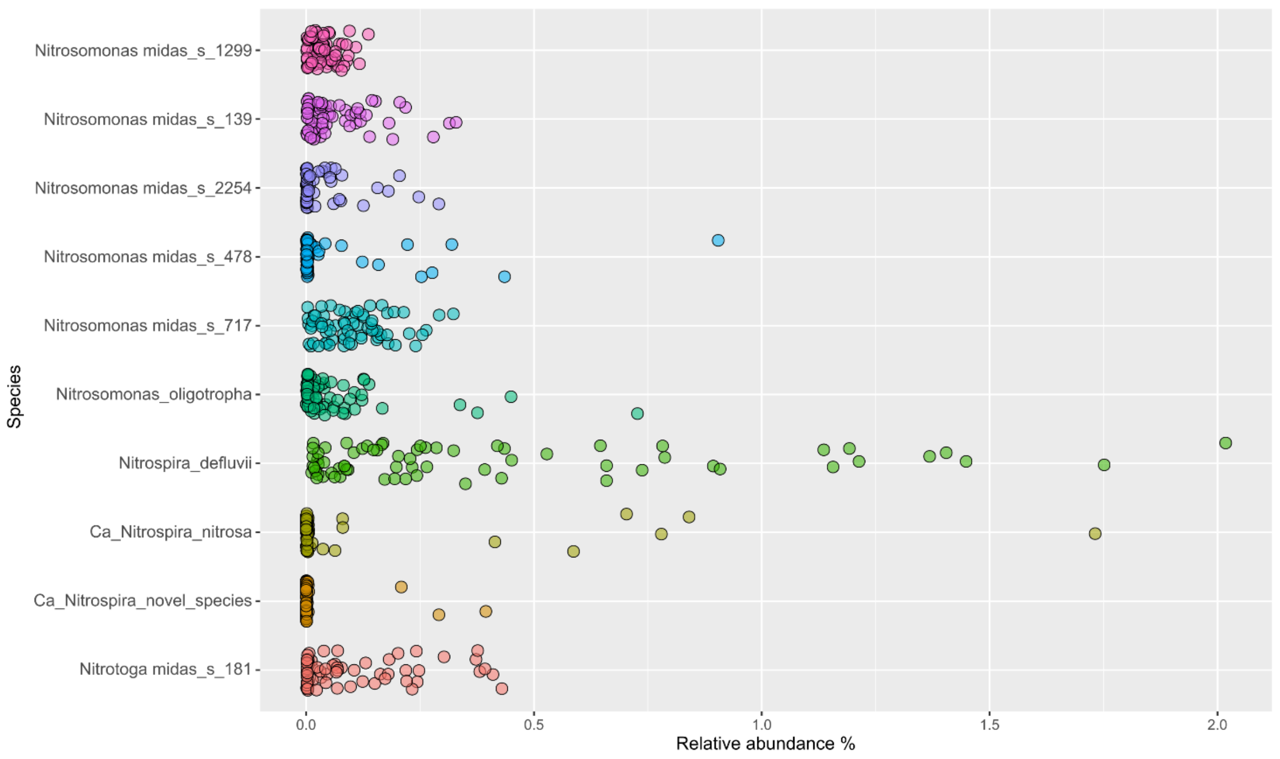

Supplementary Fig. 5: **Average relative read abundances of nitrifiers in each of the Danish WWTPs metagenomes**. Sample size n = 69, data points from Supplementary Data 6, using MAG ID to MiDAS3 species assignment in Supplementary Data 3. Colours indicate the different species.

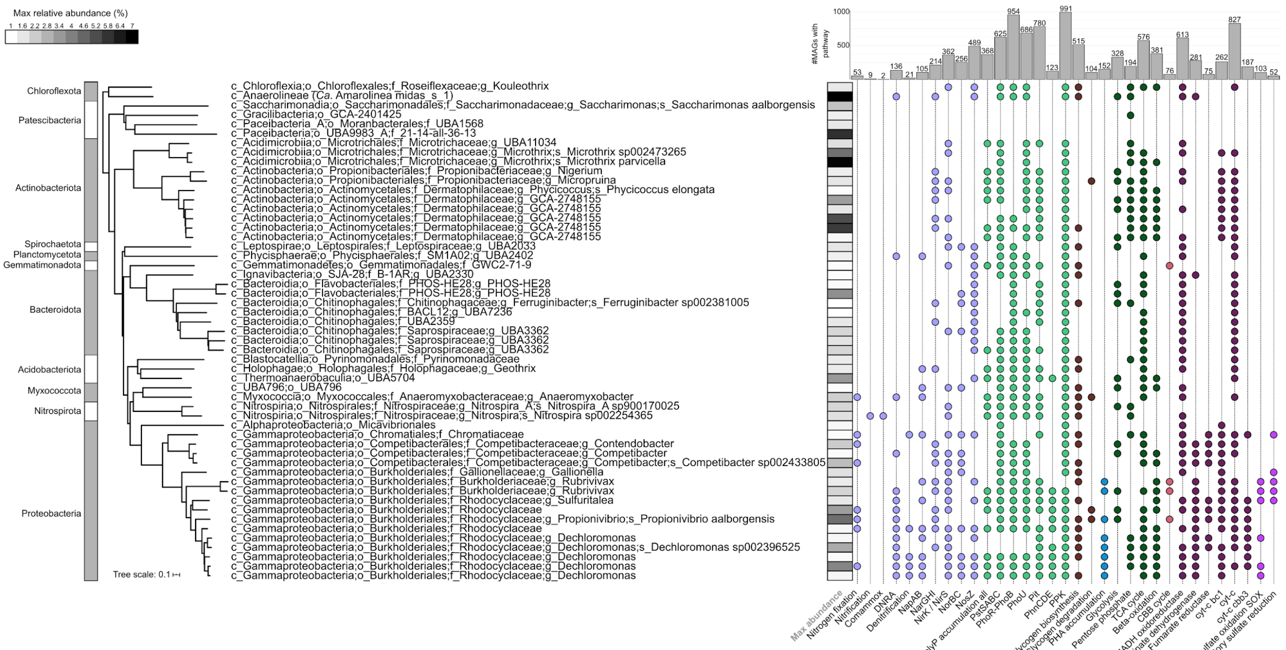

Supplementary Fig. 6: Functional profiles of the top 53 bacterial species representatives with relative abundances >1% in at least one sample metagenome used in this study. Taxonomy strings from GTDB are shown. Pathways are considered present if 100% of the genes in the KEGG module, or custom module (**Supplementary Data 12**), are encoded. Heatmap strip indicates the maximum relative abundance of the population in the metagenomes. Bar chart indicates the number of MAGs encoding the pathway of interest.

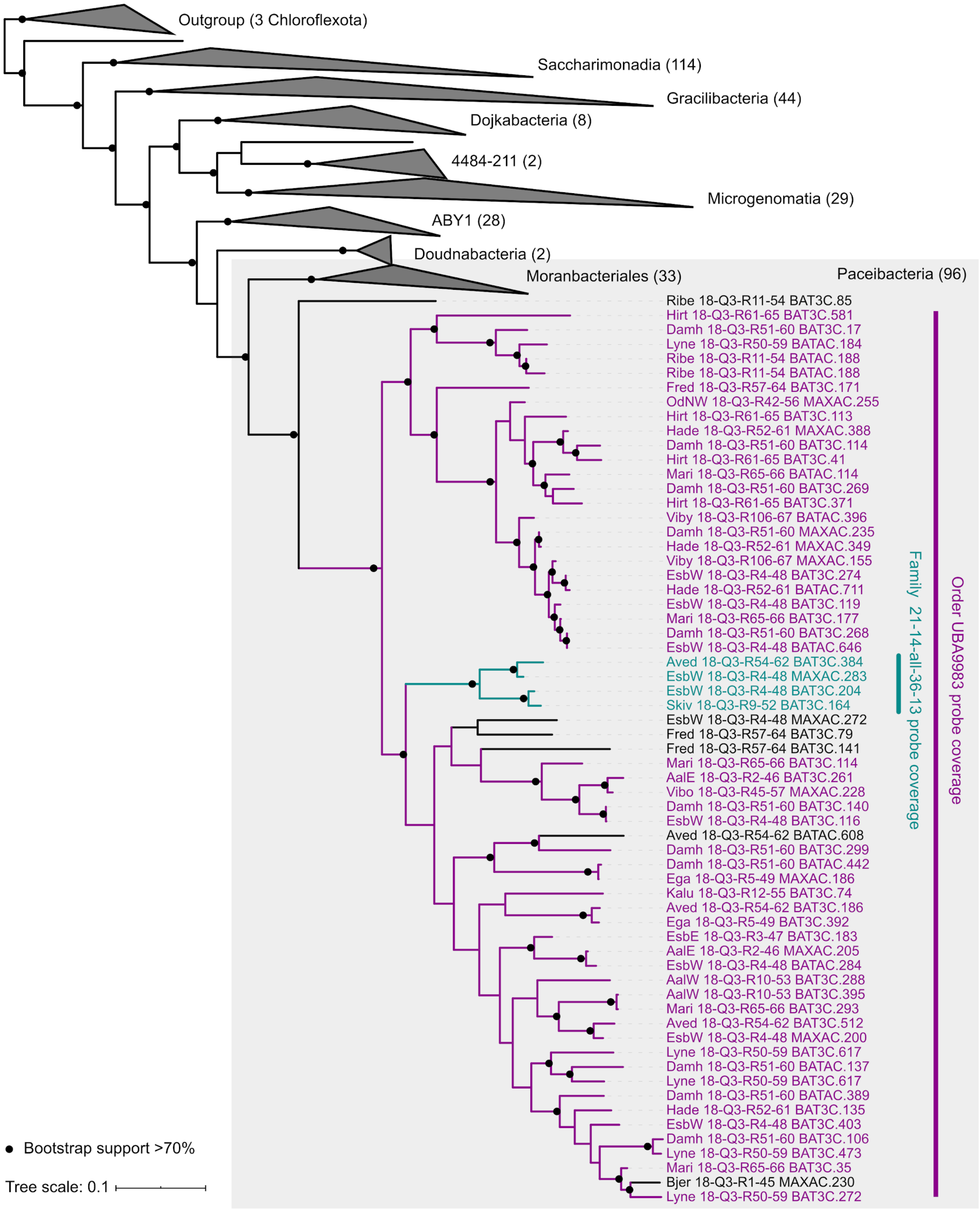

Supplementary Fig. 7: **Maximum likelihood phylogenetic 16S rRNA gene tree of Patescibacteria MAG sequences**. The coverage of the two probes used in the FISH analyses is shown, and represented by the colours. MAGs in black are missed by the probe set. The family probe (magenta) and order probe (cyan) overlap. Tree was bootstrapped 100x.

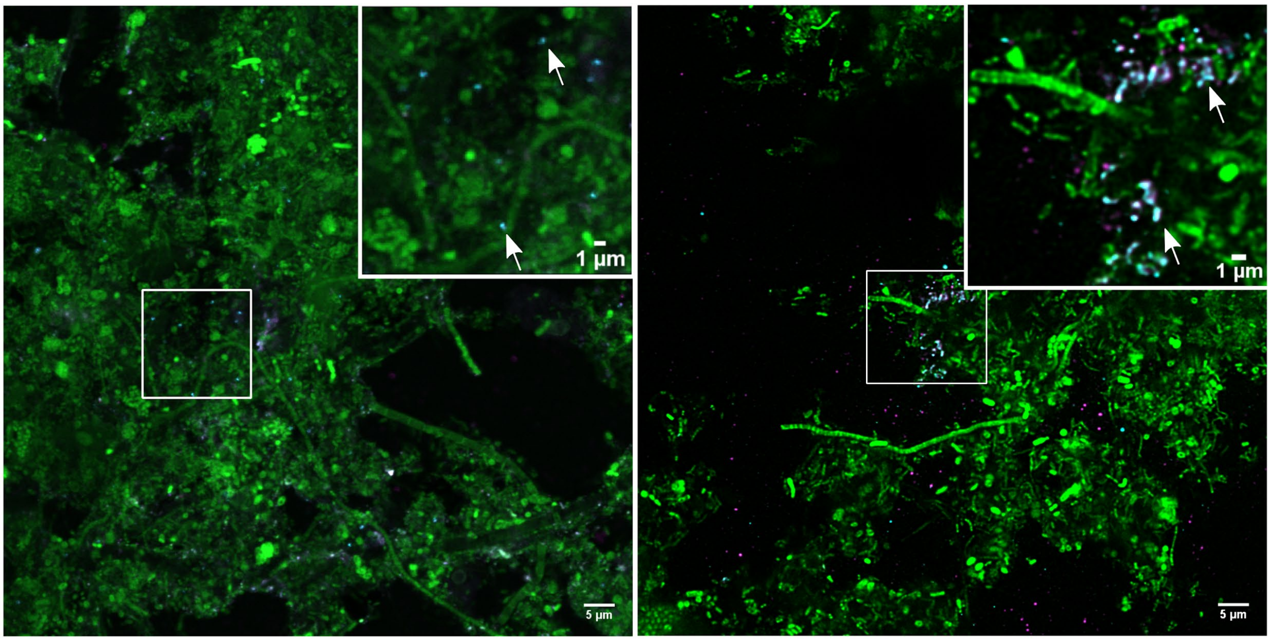

Supplementary Fig. 8: FISH images showing the ultra-small (<0.2 µm) Paceibacteria lineage. Cyan indicates the 'family level' probe (ATTO 532) and magenta the 'order level' probe (ATTO 594), and green indicates the other bacteria (EUB mix ATTO 633). Paceibacteria were very abundant (>7% based on metagenome relative abundance) and found as dispersed cells in the Esbjerg W (2018) AS sample (left), and localised cell clusters in lower abundance (0.7%) in the Viborg (2018) AS sample (right). Scale bars indicate 5 µm. Insets show magnified areas, arrows indicate cells, inset scale bars indicate 1 µm. Source data are provided as a Source Data File in Figshare [1].

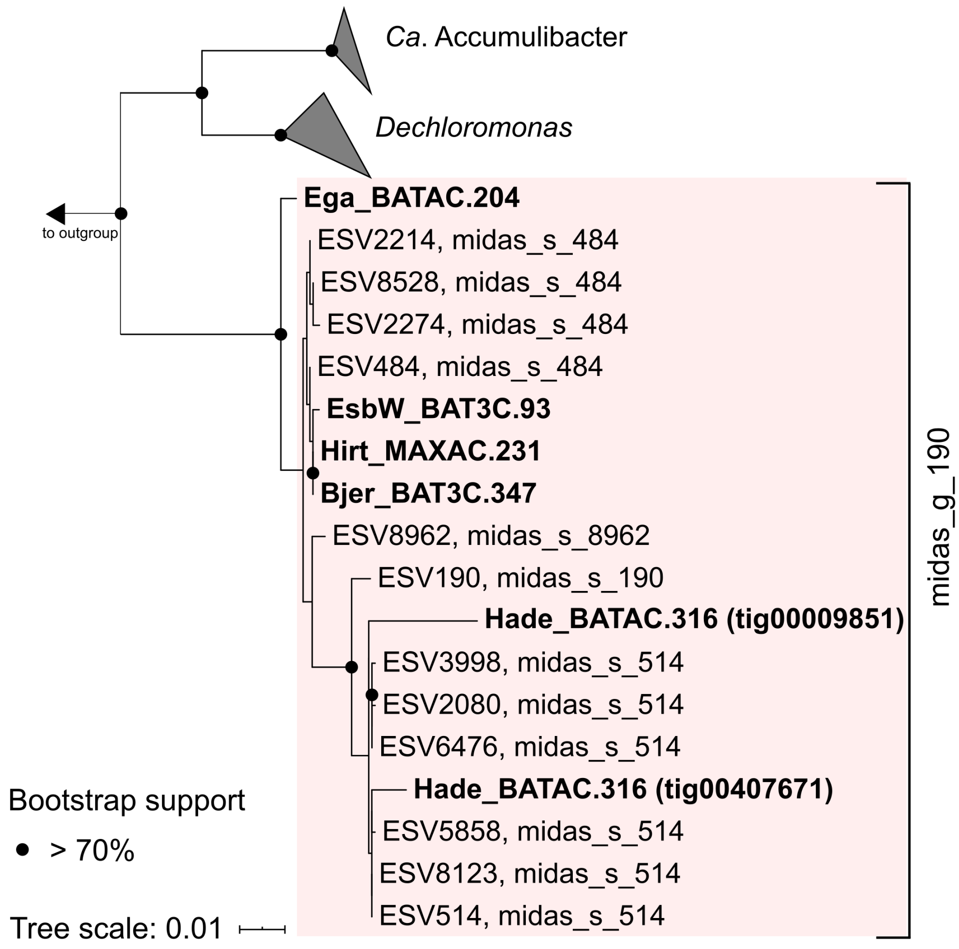

Supplementary Fig. 9: Maximum likelihood tree of the 16S rRNA gene sequences from the midas_g_190 MAGs and MiDAS3 reference database. 1000-replicate bootstraps were used. The orange box indicates coverage of the genus-specific probe g190_1276.

RESULTS : Mismatch Analysis

```
PROBE ALIGNMENT WITH TARGET ORGANISM:
................................
PROBE--3'CGCTTAGAGTGTTTCGGCTAGCAT5'
.........|||||||||||||||||||||||
TARGET-5'GCGAATCTCACAAAGCCGATCGTA3'
................................

PROBE ALIGNMENT WITH NON TARGET ORGANISM:
...........C....................
PROBE--3'CG.TTAGAGTGTTTCGGCTAGCAT5'
.........||.||||||||||||||||||||
TARGET-5'GC.AATCTCACAAAGCCGATCGTA3'
...........T....................
```

	TARGET ORGANISM	NON-TARGET ORGANISM	ΔValue(?)
$\Delta G^o{}_1$	-24.6 kcal/mol	-21.0 kcal/mol	3.60 kcal/mol
$\Delta G^o{}_2$	0.3 kcal/mol	0.3 kcal/mol	NA
$\Delta G^o{}_3$	-9.6 kcal/mol	-7.5 kcal/mol	2.10 kcal/mol
$\Delta G^o{}_{overall}$	-14.7 kcal/mol	-13.3 kcal/mol	1.40 kcal/mol
$[FA]_m$*	29.1 %	18.5 %	-10.60 %
Hybridization Efficiency**	0.9997	0.9965	-0.00

* Melting formamide concentration
** At 0% formamide

FORMAMIDE CURVE

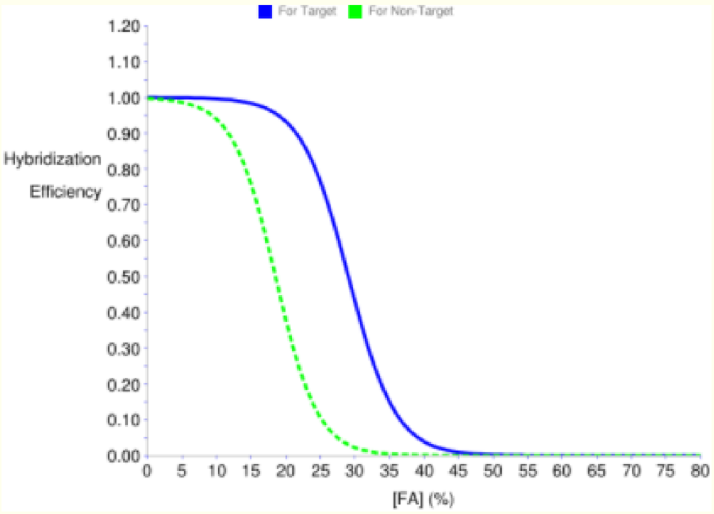

Supplementary Fig. 10: mathFISH curve for the probe g190_1276 without competitor probe. Target organism is EVS190, non-target organisms is ESV215.

RESULTS : Competitor Analysis

*

Check: Probe is a perfect match to target, and has 1 mismatches to non-target.
Competitor is a perfect match to non-target and has 1 mismatches to target

	Probe - Target	Probe - Non Target	Competitor - Target	Competitor - Non Target
**ΔG^{o}_{1} kcal/mol**	-24.6	-21.03	-21.42	-23.25
**ΔG^{o}_{2} kcal/mol**	0.32	0.32	0.32	0.32
**ΔG^{o}_{3} kcal/mol**	-9.56	-7.48	-9.56	-7.48
**$\Delta G^{o}_{overall}$ kcal/mol**	-14.74	-13.25	-11.56	-15.47
**Hybridization Efficiency***	1.0	1.0	0.95	1.0

***At 0% formamide**

FORMAMIDE CURVE

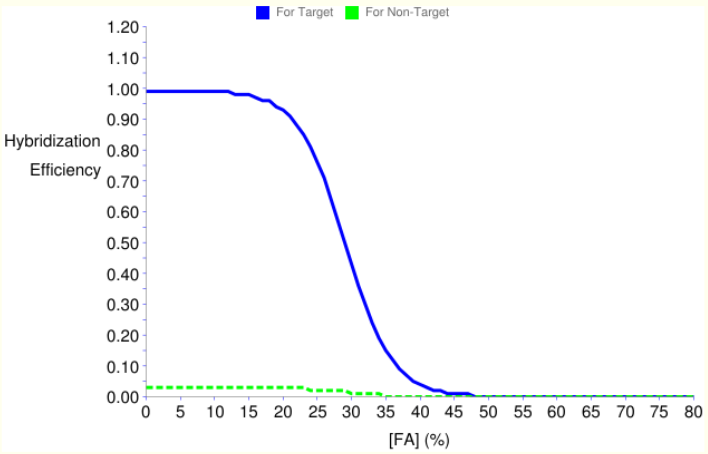

Supplementary Fig. 11: mathFISH curve for the probe g190_1276 with competitor probe. Target organism is ESV190, non-target organisms is ESV215.

Supplementary Tables

Supplementary Table 1: Summary of FISH gene probes used for Paceibacteria and *Ca*. Methylophosphatis (midas_g_190). Coverage for Paceibacteria 16S rRNA gene sequences is from the genomes only, as the target group is not available in MiDAS3. Only 1 non-target hit for Pac_683 was determined in MiDAS3. Pac_683 targets 508 sequences in SILVA v138. 444 of these sequences are from within the Parcubacteria, the remaining non-target bacteria hits are within the Patescibacteria (Candidate Phyla Radiation). Pac_133 has 0 non-target hits in SILVA v138, and targets one sequence from the Cambellbacteria. Pac_113 was used with ATTO 532, Pac_683 in ATTO 594 and EUBmix in ATTO 633 to ensure no crosstalk between the dyes.

Probe	*E. coli* pos.	Target group	Coverage	Non-target hits	Sequence (5'-3')	[FA] %	Reference
Pac_113	113-134	Family "21-14-all-36-13"	4/4	0	ACG TTC CCA CCT GTT ACT ACC	25	This study
Pac_683	683-705	Class "UBA9983"	57/63	1	TCA ACG GAT TTC ACC CCT ACA C	25	This study
g190_1276	1276-1300	Midas_g_190	12/12	0	TAC GAT CGG CTT TGT GAG ATT CGC	35	This study
g190_1276_C1	1276-1300	Competitor for g190_1276 probe	N/A	N/A	TAC GAT CGG CTT TGT GAG ATT RGC	N/A	This study
EUB338-I<br>EUB338-II<br>EUB338III	338-355<br>338-355<br>338-355	Most bacteria	N/A	N/A	GCT GCC TCC CGT AGG AGT<br>GCA GCC ACC CGT AGG TGT<br>GCT GCC ACC CGT AGG TGT	N/A	(Amann et al., 1990; Daims et al., 1999)
non-EUB		Negative control for hybridization	N/A	N/A	ACT CCT ACG GGA GGC AGC	N/A	(Wallner et al., 1993)

Supplementary Table 2: Summary of amplicon sequencing and qFISH relative abundances of *Ca*. Methylophosphatis (midas_g_190). At least 30 different FISH images were recorded from each sample for the quantification.

WWTP	Sample date	Abundance (%)	
		Sequencing	qFISH
**Midas_g_190**			
Bjergmarken	August 2018	1	2.2 ± 0.7
Hjørring	August 2015	1	1.2 ± 0.6
Ribe	August 2013	2.3	2.3 ± 0.9
Ringkøing	June 2014	1.6	2.2 ± 0.7

Supplementary Notes

Supplementary Note 1

Initially the most abundant Paceibacteria population (MAG EsbW_18-Q3-R4-48_MAXAC.283) was recovered in two contigs. Extraction and mapping of the Nanopore and Illumina reads belonging to the MAG, followed by reassembly with Flye enabled the MAG to be closed (see Methods section: Reassembly of 17 Patescibacteria additional CMAGs). Examination of the mapping data, using Tablet, and the Flye assembly graph showed that the MAG could be circularised either with, or without, a 40 kbp DNA fragment. This fragment had 50% less coverage than the rest of the genome, and was found to insert between a tandem repeat in the population genome. The 40 kbp fragment encoded 43 CDS regions as determined by Prokka v1.14, predominantly hypothetical proteins as well as phage cluster methyltransferases, DNA methylases, phage portal, peptidase and capsid proteins. Both VirSorter v1.0.5 (category 2 prophage) and PHASTER identified the insert as viral [2,3]. Only three bp were identified as potentially polymorphic (**Supplementary Data 3**), indicating the presence of a single dominant population and little strain heterogeneity. Consequently, the use of long-read sequencing enabled us to determine that half of this Paceibacteria population appears to be infected by an active phage system.

Supplementary Note 2

The *Ca.* Methylophosphatis populations encode a versatile metabolism, including the methylotrophy pathway. Based on the relatedness of this new genus to the well characterised methylotrophic genus *Methyloversatilis*, we suggest methylotrophy is a potential strategy for energy generation and/or carbon assimilation. C1 carbon dissimilation was indicated by presence of the XoxF methanol dehydrogenase in four of the five MAGs (both species), as well as the tetrahydromethanopterin pathway for formaldehyde oxidation to formate, and formate oxidation genes (**Supplementary Data 16**). C1 assimilation to acetyl-CoA was encoded by the tetrahydrofolate pathway and key genes of the serine cycle (**Supplementary Data 16**). Furthermore, carbon assimilation could also occur through the Calvin-Benson-Bassham cycle (**Figure 5**, **Supplementary Data 16**). However, unlike *Methyloversatilis* spp., the MAGs encoded no pathway for methylamine use (methylamine dehydrogenase MauA, MauB, or methylglutamate dehydrogenase MgdABCD) [4,5]. Other metabolisms encoded by the MAGs include the potential for beta-oxidation of fatty acids, glycogen degradation, fructose and acetate use (**Figure 5**, **Supplementary Data 16**). The Pit transporter was identified in all 5 MAGs, as was PhoU and the PhoR-PhoB phosphate regulatory system. The PstABCS system for phosphate transport and the PhnABC system for phosphonate transport were determined in the 4 *Ca*. M. roskildensis MAGs but not *Ca*. M. haderslevensis. In contrast, *Ca*. M. haderslevensis encoded the full pathway for PHA storage (PhaABCZ), whereas the *Ca*. M. roskildensis MAGs all missed the *phaB* gene. Nitrogen metabolism also differed between the two species. Nitrogen fixation and respiratory nitrate reduction to nitrite was present in *Ca*. M. roskildensis, but not *Ca*. M. haderslevensis which had periplasmic nitrate reductase (NapAB) and encoded nitrite reduction to nitric oxide and onto nitrous oxide (**Figure 5**). This suggests different roles or niches for the two species.

Supplementary References

1. Singleton, C. *et al.* Supplementary Dataset Associated with 'Connecting structure to function with the recovery of over 1000 high-quality metagenome-assembled genomes from activated sludge using long-read sequencing'. (2021) doi:10.6084/M9.FIGSHARE.C.5277035.
2. Roux, S., Enault, F., Hurwitz, B. L. & Sullivan, M. B. VirSorter: mining viral signal from microbial genomic data. *PeerJ* **3**, e985 (2015).
3. Arndt, D., Marcu, A., Liang, Y. & Wishart, D. S. PHAST, PHASTER and PHASTEST: Tools for finding prophage in bacterial genomes. *Brief. Bioinform.* **20**, 1560–1567 (2019).
4. Smalley, N. E. *et al.* Functional and genomic diversity of methylotrophic Rhodocyclaceae: description of Methyloversatilis discipulorum sp. nov. *Int. J. Syst. Evol. Microbiol.* **65**, 2227–2233 (2015).
5. Latypova, E. *et al.* Genetics of the glutamate-mediated methylamine utilization pathway in the facultative methylotrophic beta-proteobacterium Methyloversatilis universalis FAM5. *Mol. Microbiol.* **75**, 426–439 (2010).
