# Supplementary material for: Connecting structure to function with the recovery of over 1000 high-quality metagenome-assembled genomes from activated sludge using long-read sequencing
Source: Nat Commun. 2021 Mar 31;12:2009. doi: 10.1038/s41467-021-22203-2 (PMC8012365; doi:10.1038/s41467-021-22203-2)
Supplement: Supplementary file 2 — Description of Additional Supplementary Files [file 41467_2021_22203_MOESM2_ESM.pdf]

Description of Additional Supplementary Files

File Name: Supplementary Data 1
Description: Data yield for each metagenome and associated sample metadata

File Name: Supplementary Data 2
Description: Flow cell yield and summary statistics for the PromethION run

File Name: Supplementary Data 3
Description: MAG summary statistics. See Methods for details regarding the contents of each column. The column descriptions follow the same order as the Supplementary Data 3 file and are as follows: MAG = MAG ID, NCBI_accession_number = GenBank accession for the MAG assembly, NumContigs = Number of contigs in the MAG, TotBP = total base pairs of the MAG (genome size), MaxContigBP = length of the longest contig, AvContigBP = average contig length, HQMAG = part of the HQ MAG set, HQdRep = species level clusters from dRep at 95% ANI, HQdRep99 = near genome duplicate level clusters from dRep at 99% ANI, HQSpRep = MAG is the best quality species at 95% ANI (SPREPHQ) or not (NA), Comp = completeness from CheckM, Cont = contamination from CheckM, StrHet = strain heterogeneity from CheckM, Circ = circular MAG as identified by CANU or Flye and Unicycler assembly graphs, FLSSU = number of full-length 16S rRNA genes, FLLSU = number of full-length 23S rRNA genes, GTDBTax = genome taxonomy database classification, MiDAS3_7 = MiDAS3.7 database taxonomy assigned to 16S rRNA gene by USEARCH, MIdentity = identity to the MiDAS3.7 sequence, MAlnLgth = the alignment length to the MiDAS3.7 sequence, SILVA138Tax = SILVA taxonomy of the 16S rRNA gene assigned my USEARCH, SIdentity = identity to the SILVA138 sequence, SAlnLgth = the alignment length to the SILVA138 sequence, ilmcov = Illumina coverage of the MAG as determined using CoverM, npcov = Nanopore coverage of the MAG as determined using CoverM, max_abundance = maximum abundance of the MAG across the metagenomes, mm27f = mismatches in the 27f primer as determined using primerprospector, mm534r = mismatches in the 534r primer as determined using primer prospector, ttl_polymorphic_rate = total polymorphic rate across the genome using cmseq, polymut_rate = polymorphic rate across the CDS regions determined using cmseq.

File Name: Supplementary Data 4
Description: Single copy markers in MAGs and assemblies identified using SingleM, for the 23 samples used for the individual sample assemblies.

File Name: Supplementary Data 5
Description: Coverage based abundances (CoverM) of MAGs across all 69 metagenomes.

File Name: Supplementary Data 6
Description: Relative abundances of the 581 species representatives across the 69 Illumina metagenomes.

File Name: Supplementary Data 7
Description: Redundant single copy ribosomal protein marker genes in envOPS12.

File Name: Supplementary Data 8
Description: Ye et al. 2020 MAG completeness and contamination results from CheckM.

File Name: Supplementary Data 9
Description: Identified Ye et al. 2020 MAG full length rRNA genes.

File Name: Supplementary Data 10
Description: Ye et al. 2020 MAG dRep species dereplication with the MAGs from this study.

File Name: Supplementary Data 11
Description: 100% complete KEGG module paths and associated KOs from EnrichM.

File Name: Supplementary Data 12
Description: Custom modules used for EnrichM analysis, and pathways used in Figure 4.

File Name: Supplementary Data 13
Description: Relative abundances (CoverM) of medium quality MAGs across all 69 metagenomes.

File Name: Supplementary Data 14
Description: Top 100 MiDAS3 species to MAGs.

File Name: Supplementary Data 15
Description: Top 100 MiDAS3 ASVs to MAGs.

File Name: Supplementary Data 16
Description: Specific KO hits for *Ca*. Methylophosphatis metabolic reconstruction.
