# Supplementary material for: Connecting structure to function with the recovery of over 1000 high-quality metagenome-assembled genomes from activated sludge using long-read sequencing
Source: Nat Commun. 2021 Mar 31;12:2009. doi: 10.1038/s41467-021-22203-2 (PMC8012365; doi:10.1038/s41467-021-22203-2)
Supplement: Supplementary file 19 — Reporting Summary [file 41467_2021_22203_MOESM19_ESM.pdf]

nature research

Corresponding author(s): Mads Albertsen

Last updated by author(s): Jan 27, 2021

Reporting Summary

Nature Research wishes to improve the reproducibility of the work that we publish. This form provides structure for consistency and transparency in reporting. For further information on Nature Research policies, see our Editorial Policies and the Editorial Policy Checklist.

Statistics

For all statistical analyses, confirm that the following items are present in the figure legend, table legend, main text, or Methods section.

n/a	Confirmed	
☐	☒	The exact sample size (*n*) for each experimental group/condition, given as a discrete number and unit of measurement
☒	☐	A statement on whether measurements were taken from distinct samples or whether the same sample was measured repeatedly
☒	☐	The statistical test(s) used AND whether they are one- or two-sided<br>*Only common tests should be described solely by name; describe more complex techniques in the Methods section.*
☒	☐	A description of all covariates tested
☒	☐	A description of any assumptions or corrections, such as tests of normality and adjustment for multiple comparisons
☒	☐	A full description of the statistical parameters including central tendency (e.g. means) or other basic estimates (e.g. regression coefficient) AND variation (e.g. standard deviation) or associated estimates of uncertainty (e.g. confidence intervals)
☒	☐	For null hypothesis testing, the test statistic (e.g. *F*, *t*, *r*) with confidence intervals, effect sizes, degrees of freedom and *P* value noted<br>*Give P values as exact values whenever suitable.*
☒	☐	For Bayesian analysis, information on the choice of priors and Markov chain Monte Carlo settings
☒	☐	For hierarchical and complex designs, identification of the appropriate level for tests and full reporting of outcomes
☒	☐	Estimates of effect sizes (e.g. Cohen's *d*, Pearson's *r*), indicating how they were calculated

Our web collection on statistics for biologists contains articles on many of the points above.

Software and code

Policy information about availability of computer code

Data collection	No software was used
Data analysis	All software that was used is described in the methods. Software is as follows: Guppy v2.2.3, qcat v1.0.1, MinionQC v1.4.0, Porechop v0.2.3, Filtlong v0.2.0, FastQC v0.11.7, MultiQC v1.7, Cutadapt v1.16, GNU parallel v20190122, CANU v1.8, MUMmer v3.2.3, R v3.5.0-3.5.2, seqtk v1.3-r106, Racon v1.3.3, Minimap2 v2.15-16, medaka v0.6.5, mmlong v0.1.2, MetaBat2 v2.12.1, MaxBin v2.2.7, FragGeneScan v1.31, Kaiju v1.6.0, MOTHUR v2.7.14, DASTool v1.1.1, CheckM v1.0.11, mmgenome2 v2.0.7, dRep v2.3.2, Flye v2.6, Unicycler v0.4.6, Bandage v0.8.1, Tablet, CMSeq, GTDN-Tk v0.3.2, pplacer v1.1, FastANI v1.2, Prodigal v2.6.2, FastTree 2 v1.2, HMMER v3.1b2, ARB v6.0.3-6, IToL v5.5, Pavian, Prokka v1.14, Infernal v1.1.2, BEDTools v2.27, Fxtract v2.3, USEARCH v11, EnrichM v0.5.0, Diamond v0.9.22, CoverM v0.3.2, SingleM v0.12.1, ampvis2 v2.5.8, SINA, IQ-TREE v1.5.6, Daime, LabSpec v6.4., Samtools v1.9, MAFFT v7.402, PrimerProspector v1.0.1, TrimAL v1.4.rev15.

For manuscripts utilizing custom algorithms or software that are central to the research but not yet described in published literature, software must be made available to editors and reviewers. We strongly encourage code deposition in a community repository (e.g. GitHub). See the Nature Research guidelines for submitting code & software for further information.

Data

Policy information about availability of data

All manuscripts must include a data availability statement. This statement should provide the following information, where applicable:

- Accession codes, unique identifiers, or web links for publicly available datasets
- A list of figures that have associated raw data
- A description of any restrictions on data availability

Data generated and used in this study, Illumina and Oxford Nanopore metagenomes and HQ MAGs, are deposited in the NCBI SRA and GenBank databases under the bioproject accession number PRJNA629478 (https://www.ncbi.nlm.nih.gov/bioproject/prjna629478). Accession numbers for the MAGs are provided in

Supplementary Data 3. Both MQ and HQ MAGs have been deposited in Figshare to enable bulk download under DOI 10.6084/m9.figshare.c.5277035 98. Source Data for the FISH images (raw TIFF format) are also available in the Figshare collection. Data yield and MAG statistics are presented in the Supplementary Data Files .The EnrichM v10 database, including the KO annotated uniref100 database is found at https://data.ace.uq.edu.au/public/enrichm/. GTDBTk Refseq release 89 database is found at https://data.ace.uq.edu.au/public/gtdbtk/release_89/. The Kaiju proGenomes database is found at http://kaiju.binf.ku.dk/server. The MiDAS 3 database is found at https://www.midasfieldguide.org/guide/downloads. SILVA v132 and v138 are found at https://www.arb-silva.de/download/archive/.

Field-specific reporting

Please select the one below that is the best fit for your research. If you are not sure, read the appropriate sections before making your selection.

☐ Life sciences ☐ Behavioural & social sciences ☒ Ecological, evolutionary & environmental sciences

For a reference copy of the document with all sections, see nature.com/documents/nr-reporting-summary-flat.pdf

Ecological, evolutionary & environmental sciences study design

All studies must disclose on these points even when the disclosure is negative.

Study description	The purpose of this study was to produce high-quality metagenome-assembled genomes from the activated sludge of 23 wastewater treatments plants across Denmark.
Research sample	The samples used were from activated sludge from 2016, 2017 and 2018. Samples are representative of the activated sludge microbial community, which is a complex collection of microbial populations. Samples were chosen to complement the MiDAS3 dataset, matching up with the same biological samples taken in the summer quarters of 2016, 2017 and 2018.
Sampling strategy	The number of samples was not subject to a sample-size calculation as the samples were used for genome recovery and not statistical investigations. 23 samples from 23 different Danish wastewater treatment plants were selected for nanopore sequencing. These samples were chosen at the wastewater treatment plants had a breadth of historical amplicon data in the MiDAS 3 database, and were representative of the activated sludge community in Denmark. An additional 46 samples were chosen from the MiDAS DNA extractions, to provide differing abundances of activated sludge microbes in order to aid differential coverage binning. The sequencing depth and breadth of the samples determined the number of genomes recovered, aided greatly by software advances.
Data collection	Samples were collected from the aeration tanks of the wastewater treatment plants by trained plant technicians, as part of the MiDAS collection, and sent overnight to Aalborg University for processing and DNA extraction. Long-read data was produced by the authors at the Center for Microbial Communities using the Oxford Nanopore PromethION. Short-read data was produced by Admera Health. Data was analysed at the Center for Microbial Communities by the authors.
Timing and spatial scale	Samples were collected as part of the MiDAS program, with sampling occurring four times a year over the past 13 years. The samples used in this manuscript were from summer (quarter 3), and were collected from the activated sludge aeration tanks. The metadata associated with each sample, including sample collection date, are described in the supplementary data files and in the SRA database. Sampling was conducted by plant technicians at one time point per summer quarter.
Data exclusions	All metagenomes were used in the study.
Reproducibility	The work is observational and discovery based, consequently reproducibility was not relevant to the study aims. However, the MAG data can be reproduced following the pipeline described in the paper.
Randomization	We did not allocate experimental groups, categories are based on the sample origin which is in this case the wastewater treatment plants.
Blinding	Due to the discovery and insight nature of the work, blinding was not determined to be relevant.

Did the study involve field work? ☐ Yes ☒ No

Reporting for specific materials, systems and methods

We require information from authors about some types of materials, experimental systems and methods used in many studies. Here, indicate whether each material, system or method listed is relevant to your study. If you are not sure if a list item applies to your research, read the appropriate section before selecting a response.

Materials & experimental systems

n/a	Involved in the study
☒	☐ Antibodies
☒	☐ Eukaryotic cell lines
☒	☐ Palaeontology and archaeology
☒	☐ Animals and other organisms
☒	☐ Human research participants
☒	☐ Clinical data
☒	☐ Dual use research of concern

Methods

n/a	Involved in the study
☒	☐ ChIP-seq
☒	☐ Flow cytometry
☒	☐ MRI-based neuroimaging
